# Probing Electrocatalyst Design for Product Selectivity in CO_2_ Reduction

**DOI:** 10.1002/advs.76718

**Published:** 2026-07-24

**Authors:** Suvodeep Sen, Bapan Biswas, Apinya Ngoipala, Julia Wiktor, Ujjwal Pal, Shalini Singh

**Affiliations:** ^1^ Department of Chemical Sciences and Bernal Institute University of Limerick Limerick Ireland; ^2^ Department of Energy & Environmental Engineering CSIR Indian Institute of Chemical Technology Hyderabad Telangana India; ^3^ Academy of Scientific & Innovative Research (AcSIR) Ghaziabad Uttar Pradesh India; ^4^ Department of Physics and Astronomy Chalmers University of Technology Gothenburg Sweden

**Keywords:** CO_2_ reduction, colloidal nanocrystals, electrocatalyst design, product selectivity

## Abstract

Electrochemical CO_2_ mitigation offers a transformative route to sustainability, offering a powerful solution to the twin challenges of rising global energy demand and climate change. The formation of multicarbon products, particularly hydrocarbons and oxygenates, is of considerable industrial significance owing to their higher energy density and value as key chemical feedstocks. Despite significant progress, pivotal challenges persist in optimizing selectivity, streamlining reaction pathways, and enabling scalable implementation. This review outlines recent progress in the rational design of electrocatalysts for the selective CO_2_ reduction reaction (CO_2_RR), emphasizing operational conditions and strategies that manipulate the local reaction microenvironment to steer selectivity and efficiency. It presents an in‐depth discussion of the stability of Cu‐ and non‐Cu‐based electrocatalysts, including single‐atom and molecular catalysts, and their degradation mechanisms, with both ex/in situ analysis and computational insights. Special attention is given to structure‐performance relationships and dynamic surface reconstructions under reaction conditions. Finally, it sheds light on promising pathways for achieving highly selective formation of higher‐order hydrocarbons (C_3_−C_6+_). The review concludes by identifying pressing challenges and highlighting emerging frontiers in CO_2_RR that are poised to accelerate progress. These insights collectively chart a course for translating CO_2_R research into viable industrial applications, thereby advancing global efforts toward carbon neutrality.

## Graphical abstract

Selective CO_2_ electroreduction is controlled by catalyst structure, dynamic surface reconstruction, adsorbate interactions, and reaction microenvironment. This review summarizes Cu‐ and non‐Cu‐based catalysts, single‐atom and molecular systems, degradation pathways, and operando/computational strategies for steering CO_2_RR toward hydrocarbons, oxygenates, and higher‐order multicarbon products.

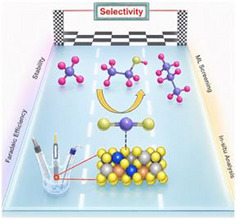

## 1 Introduction

Energy constitutes an indispensable cornerstone of human civilization, fueling every facet of modern life. However, the relentless and unsustainable exploitation of non‐renewable resources has unleashed a catastrophic surge in global warming and a drastic depletion of Earth's finite energy reserves [1]. At the epicentre of this environmental crisis lies the soaring concentration of carbon dioxide (CO_2_), a dominant greenhouse gas, intensified by the continuous release of toxic pollutants from the widespread combustion of carbon‐based fossil fuels [2]. This unbridled surge in CO_2_ emissions represents a profound and escalating threat to human health, ecological integrity, and socioeconomic fortitude. The consequences are far‐reaching, compelling an imperative mandate for a swift decarbonization strategy, to preserve our planet and secure a habitable, just future for generations. A successful transition hinges on a comprehensive strategy that includes an accelerated exodus from fossil fuel dependency towards inexhaustible renewable energy sources, [3, 4, 5, 6] such as wind, [7] solar, [8, 9, 10, 11, 12, 13] and hydropower, coupled with revolutionary leaps in energy efficiency that permeate every sector. However, eradicating fossil fuels confronts formidable barriers, rooted in our ingrained dependence on carbon‐derived essentials [14]. Deploying carbon capture, utilization, and storage technologies at scale is becoming essential to global efforts to reduce CO_2_ emissions [15, 16]. While CO_2_ capture and geological sequestration demand substantial energy input, repurposing CO_2_ as a feedstock for the synthesis of high‐value chemicals and fuels presents a compelling alternative [17, 18, 19, 20]. This paradigm not only mitigates greenhouse gas emissions but ignites a circular carbon economy, converting waste into boundless wealth, while confronting head‐on the planet's most pressing energy and environmental challenges. CO_2_, repurposed as a versatile feedstock, yields an arsenal of high‐value products, including formic acid (HCOOH), methane (CH_4_), formaldehyde (HCHO), methanol (CH_3_OH), and a range of multi‐carbon compounds that serve as critical intermediates in the chemical and energy industries. Despite significant progress, key challenges in CO_2_RR are the lack of product selectivity, which often leads to the simultaneous formation of a wide range of hydrocarbons and oxygenates rather than a single desired product. This non‐selective behavior complicates downstream separation and reduces overall process efficiency. In addition, maintaining long‐term catalytic stability remains a major hurdle, as catalysts frequently undergo structural degradation, surface reconstruction, or poisoning under operating conditions. Compounding these issues, the competing hydrogen evolution reaction (HER) further diminishes CO_2_RR efficiency by diverting electrons and protons toward H_2_ formation, thereby lowering both the Faradaic efficiency and selectivity of the desired carbon‐based products.

Given its decisive influence on selectivity, stability, and sustainability, catalyst selection remains central to the success of CO_2_ electroreduction. The motivation of this review is founded on the need to understand the current advances in the electrocatalyst design for the reduction of CO_2_ to selectively form value‐added chemical products. The first section of this review provides a fundamental overview of the mechanistic aspects of the electrochemical CO_2_RR that govern both the reaction rate and product selectivity. This includes the role of thermodynamics, reaction kinetics, and microenvironment on product selectivity. Then it delves into the different types of electrocatalysts (from Cu to non‐Cu‐based systems), outlining their advantages and challenges, including selectivity, performance, restructuring, and stability. A systematic integration of operando spectroscopic techniques with advanced computational modelling, essential for establishing reliable descriptors that connect composition, molecular effects, electronic structure, and product distribution, is comprehensively discussed. It ultimately clarifies which reaction routes are most effective for selectively producing higher‐chain hydrocarbons. Finally, the review concludes by outlining future perspectives and research directions, emphasising the theoretical and experimental approaches needed to overcome current challenges.

## 2 Thermodynamic and Kinetic Foundations of CO_2_ Reduction

**FIGURE 1:**
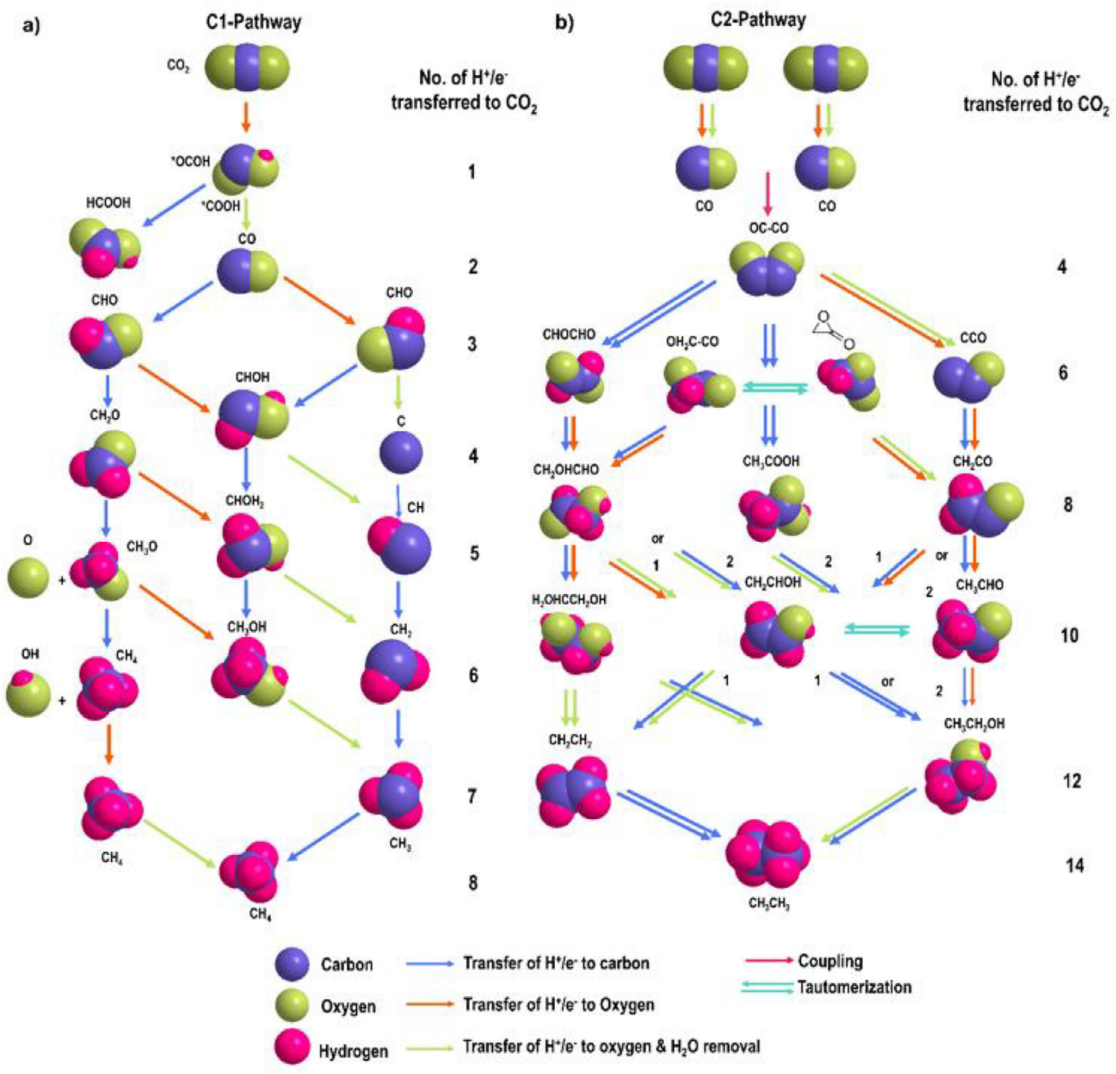
Schematic molecular model illustrating potential CO_2_RR pathways leading to the formation of (a) C_1_ and (b) C_2+_ products. The reaction routes shown here represent only a subset of the possible pathways.

CO_2_ is an amphoteric molecule [21] with a relatively high first ionization potential (∼13.8 eV) [22] and modest electron affinity (−0.66 eV), [23] making CO_2_ thermodynamically more favorable as an electron acceptor than as a donor. The reduction of CO_2_ is inherently complex, as the pronounced electrophilic nature of the carbon atom outweighs the weak nucleophilic tendencies of the oxygen atoms. This impacts the electroreduction of CO_2_, due to their multistep proton‐electron transfer pathways and the coexistence of numerous competing intermediates and products. CO_2_RR is initiated by the binding of CO_2_ to the catalyst surface, forming adsorbed *CO_2_ or *CO_2_^−^ species, which requires a highly negative reduction potential of −1.9 V [24, 25]. This step is particularly significant because it involves injecting an electron into the (C─O) π* orbital of the linear, sp‐hybridised CO_2_ molecule, forcing a symmetry‐breaking transformation to a bent sp^2^ radical anion with an O─C─O angle of 138° [26, 27]. This structural reorganisation imposes a considerable energy barrier. Furthermore, the reduction potential for H^+^ is close to that of various CO_2_RR products, leading to intense competition between proton reduction and CO_2_RR. This competition often results in diminished Faradaic efficiency (F.E.), as electrons are diverted away from CO_2_RR. A key requirement for an effective catalyst is the ability to adsorb CO_2_, thereby stabilising the transition state, lowering the kinetic barrier of the rate‐determining step, and minimising competition with proton reduction. Partial electron transfer to CO_2_ upon adsorption encourages the molecule to adopt a bent geometry and lower its lowest unoccupied molecular orbital (LUMO) level, making it more amenable to reduction and enhancing overall catalytic performance. Subsequent reaction steps proceed through sequential proton and/or electron transfer, [28] yielding a range of surface‐bound intermediates and final products. As illustrated in Figure 1a, one representative reaction network traces the conversion of CO_2_ to C_1_ products via the CO‐mediated pathway. The blue and red arrows indicate the transfer of H^+^/e^−^ to the C‐ and O‐sites of the adsorbed molecule, respectively. The blue arrows denote H^+^/e^−^ transfer to the oxygen site, followed by the subsequent removal of a water molecule. Here, the CO pathway is used as a representative example for C_1_ formation. Based on the same conceptual framework, other analogous reaction routes, such as the HCOOH pathway, can also be constructed. While C_1_ product formation is relatively straightforward, producing C_2+_ products is more complex, owing to the larger number of possible protonation sites and the potential involvement of non‐electrochemical steps. Figure 1b illustrates a plausible reaction network for the formation of C_2_ species through the *CO dimerization pathway. This is the most widely accepted pathway, leading to products such as C_2_H_6_, C_2_H_4_, C_2_H_5_OH, and n‐C_3_H_7_OH. These higher‐order products are particularly attractive due to their greater energy density and ease of transport, making them more practical as fuels and chemical feedstocks.

Transforming CO_2_ into value‐added chemicals demands an inherently endergonic process, marked by a steep positive Gibbs free energy (ΔG > 0) ascent [27]. This mandates that a substantial energy input is required to activate the chemically inert CO_2_ molecule, rendering the pathway thermodynamically uphill and challenging from both an energy and economic perspective. Although breakthroughs in the late 2000s accelerated research innovation in catalyst architecture and process refinement, a core challenge persists: discovering top‐performing catalysts that selectively generate target products. This demands rigorous scrutiny of the material's catalytic prowess, its aptitude to activate adsorbed CO_2_ molecules and facilitate their transformation. Pivotal here is the adsorption tenacity between CO_2_ and the catalyst surface, which profoundly dictates the activation efficacy.

**FIGURE 2:**
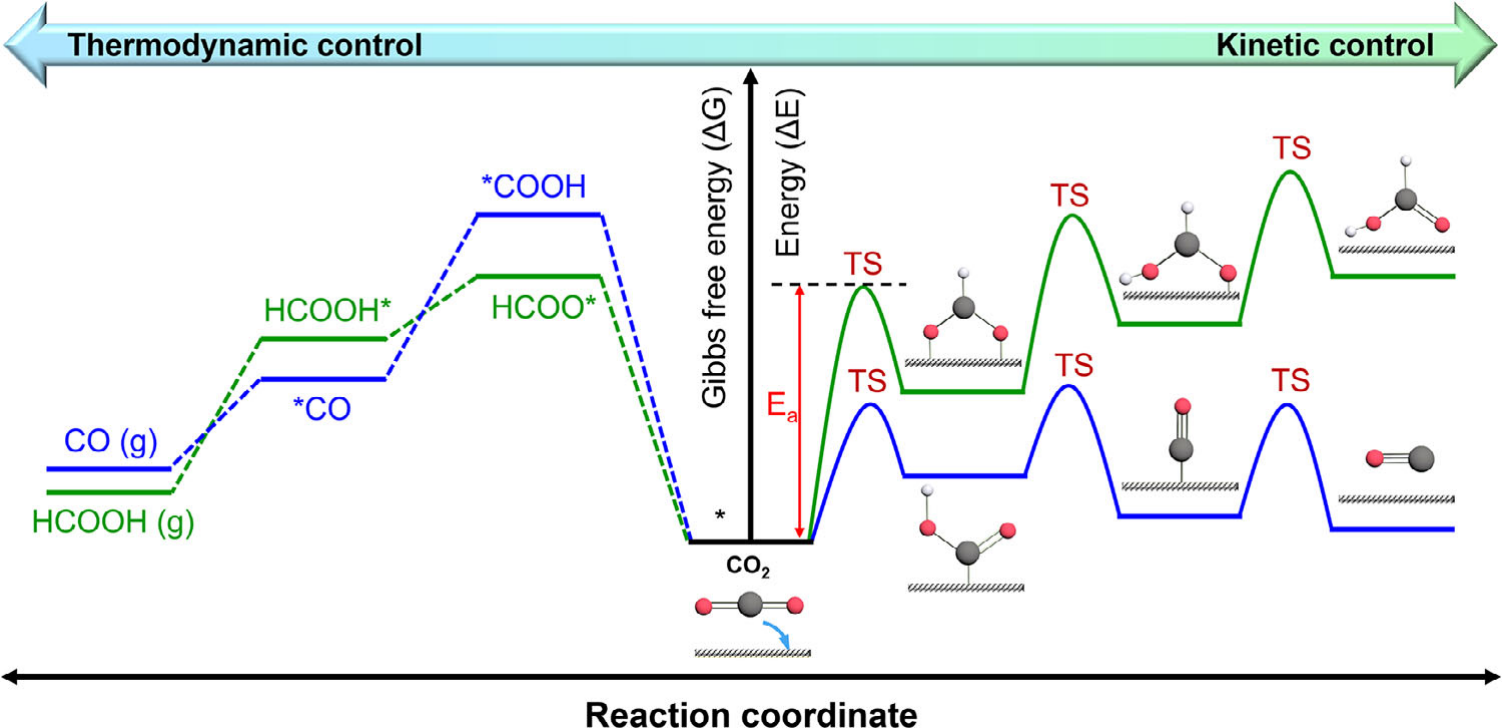
Schematic reaction energy diagrams illustrating representative pathways for CO and HCOOH formation from CO_2_. The thermodynamic landscape (ΔG) highlights relative intermediate stabilities, while the kinetic landscape (ΔE) emphasizes transition states (TS) and energy barriers (E_a_) that govern pathway selectivity. The diagrams are conceptual and intended to contrast thermodynamic and kinetic control; additional competing pathways (e.g., C─C coupling toward C_2+_ products) may arise from shared intermediates such as *CO but are not explicitly depicted.

Achieving commercial viability in CO_2_RR requires catalysts that efficiently and selectively produce desirable products with minimal energy input. Current discovery strategies often depend on time‐intensive workflows that couple ab initio and kinetic simulations with experimental synthesis and characterization (Figure 2). Thus, a comprehensive understanding of the thermodynamics and kinetics underlying the complex, multistep CO_2_RR is essential. The challenge stems from the remarkable thermodynamic stability of CO_2_, reflected in its standard Gibbs free energy of formation (‐394.4 kJ mol^−^^1^), which makes it highly resistant to reduction. This stability is primarily due to its linear molecular geometry and the presence of strong double C═O bonds, both of which contribute to the significant energy barriers that must be overcome for effective CO_2_ activation and transformation.

Several compelling pathways for valorizing CO_2_ into high‐value hydrocarbon fuels and chemical feedstocks are discussed elsewhere [29, 30, 31, 32]. Among all options, the electrochemical reduction of CO_2_ excels with over 50% selective conversion [33, 34, 35]. Based on the current state‐of‐the‐art, CO_2_RR electrocatalysts can be broadly classified into three principal categories: metallic catalysts, non‐metallic catalysts, and molecular catalysts. Depending on the catalysts used, the intermediates formed, and their microenvironments, a variety of C_1_ and higher hydrocarbon and oxygenate products can be generated from CO_2_ reduction (CO_2_R) via a series of proton‐coupled, multi‐electron‐transfer steps. However, the technical and economic viability of CO_2_ electrolysis depends on achieving high current density, low cell voltage, high selectivity, and long‐term stability. This is largely due to the complexity of multi‐proton‐coupled electron transfer cascades. Although deterministic industrial benchmarks remain uncertain, recent literature reports suggest that current density and selectivity for smaller hydrocarbons, such as ethylene, are approaching practical targets. However, further reductions in cell voltage and substantial improvements in catalyst stability are still required for industrial deployment.

## 3 Strategic Product Selection Principle: Choosing What Matters

Over the past 15 years, research in electrochemical CO2RR has expanded rapidly, leading to the development of a range of promising catalyst systems [36]. Early efforts primarily focused on increasing reaction rates and minimizing overpotentials. More recently, however, the field has increasingly focused on the complex challenge of controlling product selectivity. In this section, we outline the impact of the microenvironment on electrocatalyst interactions during CO_2_RR. Then we will examine the mechanisms of CO_2_R by identifying, capturing, and characterizing key reaction intermediates, both from a theoretical and experimental standpoint. Insights from these studies will help clarify the underlying factors governing product selectivity in CO_2_RR.

### 3.1 Microenvironment Engineering

**FIGURE 3:**
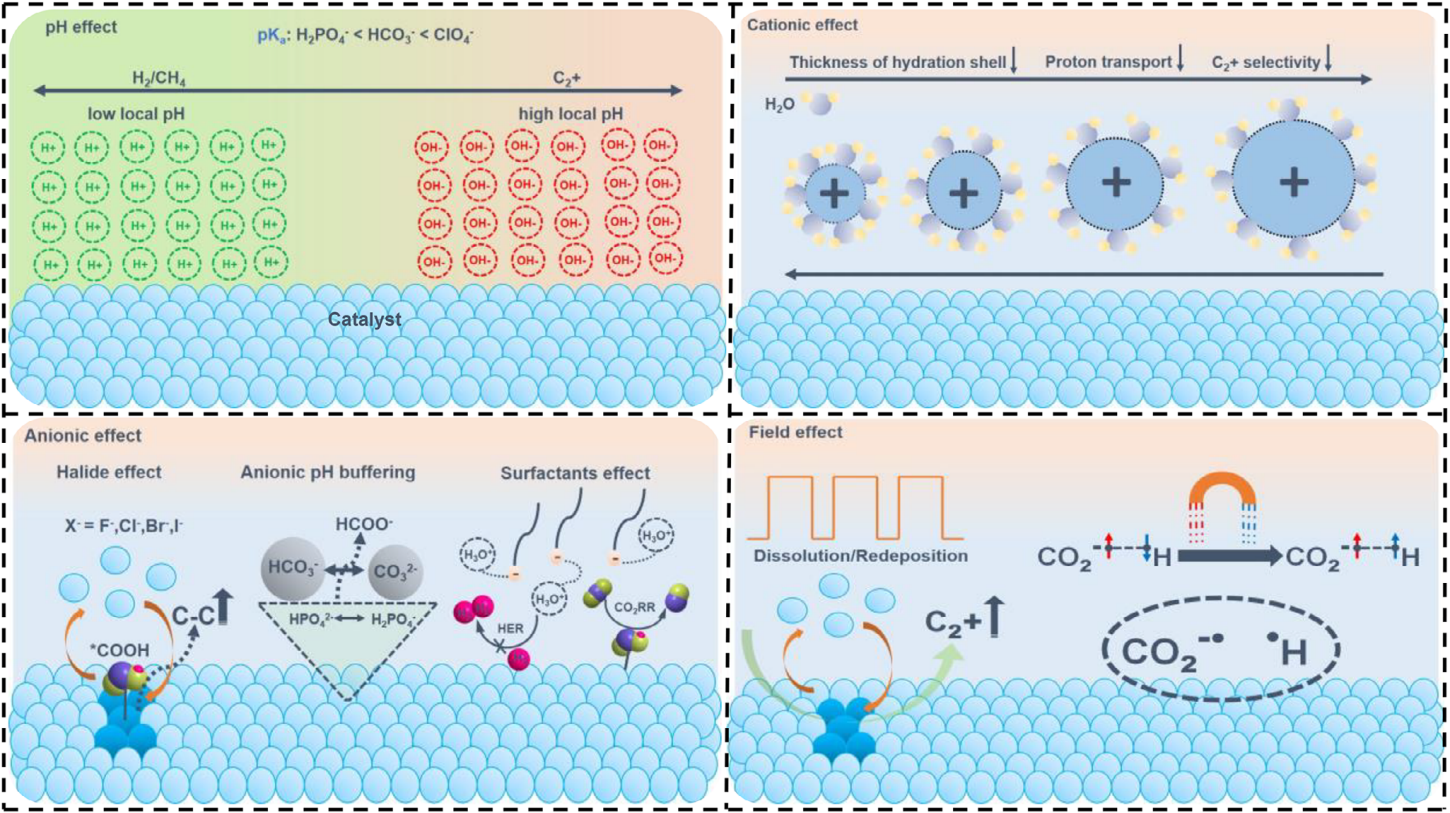
Schematic representation of micro environment engineering in electrochemical CO_2_RR.

Electrocatalytic CO_2_RR predominantly proceeds within the electrical double layer (EDL) formed at the electrode‐electrolyte interface; therefore, its efficiency is determined not only by the properties of the electrode materials (e.g., catalysts and substrates) but also by the local interfacial environment modulation. The local microenvironment can be regulated primarily through variations in interfacial electrolytes (including pH effects, ionic distributions, and the mass‐transport dynamics of reaction intermediates) and interfacial external fields. Elucidating the key factors (Figure 3) that govern the local reaction microenvironment is essential for the rational design and optimization of electrochemical interfaces. A fundamental understanding of these determinants enables precise regulation of interfacial reaction conditions, thereby enhancing reaction kinetics, product selectivity, and the overall efficiency of electrochemical conversion processes.

#### 3.1.1 pH Effect

The local pH at the electrode‐electrolyte interface exerts a greater influence on CO_2_RR performance than the bulk electrolyte pH. During CO_2_RR, proton consumption, particularly at high current densities, leads to the accumulation of hydroxide ions (OH^−^) near the electrode surface, creating a local alkaline environment that differs substantially from the bulk electrolyte [37, 38]. The selectivity of CO_2_RR reduction is highly sensitive to local pH, largely owing to variations in proton availability and transport behavior. The local pH at the cathode‐electrolyte interface increases because of OH^−^ generation during both CO_2_RR and HER. Since the rate of OH^−^ production scales with current density, the buffering capacity of the electrolyte plays a critical role in regulating local pH by determining the extent to which the generated OH^−^ species are neutralized. For instance, a 0.1 M K_2_HPO_4_ electrolyte can effectively neutralize hydroxide ions generated near the cathode surface, thereby maintaining a relatively low local pH. This interfacial environment favors the formation of H_2_ and CH_4_ while suppressing C_2_H_4_ production [39]. In contrast, non‐buffering electrolytes such as KClO_4_ and K_2_SO_4_ are unable to effectively neutralize locally generated OH^−^ ions, leading to a higher interfacial pH that promotes C_2_H_4_ formation. Consequently, using electrolytes with varying buffering capacities showed that an elevated local pH near the electrodes suppresses H_2_ and CH_4_ production while enhancing C_2_H_4_ selectivity [40, 41]. A high local pH effectively suppresses the HER by limiting proton availability and increasing the energy barrier associated with water dissociation. In alkaline environments, the scarcity of H_3_O^+^ species and the accumulation of OH^−^ ions inhibit HER kinetics, thereby reducing its competitiveness relative to CO_2_ reduction [42]. Accordingly, elevated OH^−^ concentrations generally enhance the formation of C_2__+_ products on Cu‐based catalysts by suppressing the competing HER and stabilizing key reaction intermediates, thereby improving both reaction rates and Faradaic efficiencies [43, 44, 45]. In a pioneering study, Co et al. developed an in situ rotating ring‐disk electrode approach to directly monitor local pH variations during CO_2_ reduction on Au catalysts, employing the electrogenerated CO intermediate as an intrinsic probe of the interfacial reaction environment. By transporting electrogenerated CO from the Au disk to a Pt ring electrode, the depletion of the local CO_2_/HCO_3_^−^ buffering capacity induces a Nernstian shift in the CO oxidation potential, reflecting substantial local alkalization (pH 8–10). Under identical CO_2_RR operating conditions and current densities, alkali metal bicarbonate electrolytes (MHCO_3_; M = Li^+^, Na^+^, K^+^, and Cs^+^) exhibit distinct interfacial pH environments, with the degree of local alkalization near the cathode decreasing in the order Li^+^ > Na^+^ > K^+^ > Cs^+^. These findings experimentally confirm theoretical predictions that cation‐specific electrostatic interactions at the negatively polarized electrode govern local buffering behavior and OH^−^ accumulation. Larger alkali cations, particularly Cs^+^, exhibit greater buffering capability and more effectively mitigate interfacial alkalization, whereas Li^+^ displays the weakest buffering effect [38].

However, in alkaline media, CO_2_ can react with hydroxide ions to form electrochemically inactive bicarbonate and carbonate species, reducing CO_2_ availability and thereby lowering carbon utilization efficiency [46, 47]. To address CO_2_ losses associated with carbonate formation and enhance carbon utilization efficiency during high‐rate CO_2_ electrolysis, two major strategies have been proposed: one is the regeneration and recycling of CO_2_ from carbonate species, and another is tandem catalytic schemes involving initial CO_2_‐to‐CO conversion followed by CO reduction to multicarbon products. Despite these advances, carbonate formation at cathodic gas‐diffusion electrodes remains intrinsically difficult to avoid, owing to the highly alkaline interfacial environment generated by the accumulation of OH^−^ ions during electrochemical operation in flow‐cell electrolyzers [48, 49, 50]. Acidic electrolytes have recently attracted considerable attention for CO_2_ reduction because they effectively prevent the formation of carbonate and bicarbonate species, thereby improving carbon utilization. However, the high proton concentration in acidic media also accelerates the competing HER, necessitating the development of various strategies to selectively suppress HER and enhance CO_2_RR performance. For instance, Koper and co‐workers demonstrated that under mildly acidic conditions on Au electrodes, efficient CO_2_ reduction requires sufficiently high rates of CO and OH^−^ generation at the electrode interface to effectively suppress the competing HER [51]. Sargent and co‐workers demonstrated that the incorporation of high concentrations of alkali metal cations into acidic electrolytes can modulate the interfacial pH and electric field, thereby enhancing CO_2_RR current densities. Furthermore, catalyst active‐site engineering has been employed to optimize the adsorption and stabilization of key reaction intermediates (such as *COOH, *OCOH, and *OCCO), promoting improved activity and selectivity toward desired products [37]. Despite their advantages in carbon utilization, acidic electrolytes generally exhibit lower CO_2_RR stability than alkaline systems. The high proton concentration and absence of bicarbonate/carbonate buffering species impede stable operation, while the corrosive acidic environment accelerates the dissolution and degradation of many metal and metal oxide catalysts, thereby compromising long‐term catalytic durability [52, 53, 54, 55].

#### 3.1.2 Ionic Distribution

In CO_2_RR, electrolyte‐derived cations and anions constitute key components of the interfacial microenvironment, where they play a critical role in modulating reaction pathways and product selectivity at the catalyst surface. Electrolyte cations are recognized as key regulators of the kinetics and product selectivity of electrocatalytic CO_2_ reduction. Their influence arises from interactions with the electrode surface through both specific adsorption and long‐range electrostatic effects, which collectively modulate the interfacial reaction environment and catalytic behavior [56, 57]. Within the Gouy‐Chapman‐Stern description of EDL, specifically adsorbed species are positioned at the inner Helmholtz plane, while ions interacting predominantly through electrostatic forces occupy the outer Helmholtz plane [58, 59]. The pioneering studies by Hori and co‐workers demonstrated that alkali metal cations, including Li^+^, Na^+^, K^+^, and Cs^+^, exert a significant influence on the activity and product selectivity of CO_2_ electroreduction over Cu‐based catalysts [60]. Recently, Li and co‐workers reported that the hydration characteristics of alkali metal cations are strongly dependent on ionic size within the electrical double layer. As the cation size increases from Li^+^ to Cs^+^, the degree of hydration decreases, resulting in a progressively thinner hydration shell in the order Li^+^ > Na^+^ > K^+^ > Cs^+^ [61]. Further, the Faradaic efficiency toward C_2_ products during CO_2_ electroreduction exhibits a pronounced dependence on alkali metal cation identity, increasing systematically with cation size: Li^+^ < Na^+^ < K^+^ < Rb^+^ < Cs^+^ [62, 63]. It is found that the preferential accumulation of larger alkali metal cations at the electrode‐electrolyte interface increases the positive potential at the outer Helmholtz plane, thereby reducing interfacial H_3_O^+^ concentration through electrostatic and competitive adsorption effects, suppressing proton transport while favoring CO_2_ reduction [64]. Adsorbed alkali metal cations exert minimal influence on hydrogen adsorption energetics in acidic media but can significantly promote C_2_ product formation by stabilizing key C‐C coupling intermediates [65]. In particular, larger cations facilitate the stabilization of *OCCO species and create a localized hydrophobic interfacial environment that suppresses *CO protonation, thereby enhancing C‐C coupling and favoring the production of multicarbon products over C_1_ species [66]. Further quaternary alkylammonium cations, including tetramethylammonium, tetraethylammonium, tetrapropylammonium, and tetrabutylammonium ions, have been reported to modulate the interfacial interactions between adsorbed CO intermediates and water molecules at the catalyst surface [67]. Larger quaternary alkylammonium cations weaken the interaction between surface‐adsorbed CO and interfacial water molecules, thereby suppressing CO hydrogenation pathways toward C_2__+_ products, although this cation‐dependent behavior differs in acetonitrile‐based electrolytes. In acetonitrile‐based electrolytes, larger quaternary ammonium cations (TBA^+^ > TPA^+^ > TEA^+^) exhibit a less pronounced enhancement in CO Faradaic efficiency, which has been attributed to the weaker interaction between electrode surfaces and CO intermediates compared with aqueous systems. These large cations are more effective at disrupting the interfacial organization of acetonitrile and electrolyte ions, thereby facilitating the desorption of CO intermediates from the catalyst surface. While smaller quaternary ammonium cations, such as TEA^+^, preferentially promote oxalate formation through CO_2_ radical dimerization pathways that do not involve water, increasing cation alkyl‐chain length suppresses oxalate production and shifts the reaction pathway toward CO formation via water‐assisted mechanisms [68].

Electrolyte anions play a critical role in CO_2_ electroreduction, particularly when specifically adsorbed at the electrode‐electrolyte interface. Through interactions with the electrode surface and other interfacial species, these anions can regulate local pH, induce catalyst surface restructuring, and modify the adsorption behavior of reaction intermediates. For instance, in bicarbonate‐based electrolytes, HCO_3_^−^ primarily maintains a high local CO_2_ concentration through rapid equilibrium with dissolved CO_2_ and may also serve as a supplementary CO_2_ source during electroreduction [46]. Furthermore, HCO_3_^−^ has been proposed to preferentially stabilize the *OCO intermediate rather than *COOH, thereby facilitating the formation of HCOO^−^ during CO_2_ electroreduction [69]. However, elevated bicarbonate concentrations generally promote competing H_2_ and CH_4_ formation, thereby diminishing CO_2_RR selectivity. Consequently, relatively low bicarbonate concentrations, such as 0.1 M KHCO_3_, are commonly employed to balance CO_2_ availability and product selectivity. Halide ions have been shown to enhance CO_2_ electroreduction on Cu electrodes by promoting C_2_ product formation while suppressing the competing hydrogen evolution reaction, particularly on Cu(100) facets [70]. They play a critical role in catalyst surface reconstruction, promoting the formation of highly roughened morphologies with an increased density of catalytically active sites [71]. For example, Garg et al. systematically examined the influence of halide ions in choline‐based electrolytes on the electrochemical reduction of CO_2_ to CO over Ag electrodes, revealing trends in CO Faradaic efficiency of Cl^−^ > Br^−^ > I^−^. At highly cathodic potentials, halide ions facilitate Ag dissolution and redeposition processes, leading to the formation of high‐index crystal facets, including (220), (311), and (222), which alter the catalytic surface structure and reactivity [72]. Beyond inducing catalyst surface reconstruction, halide ions facilitate the formation of the *COOH intermediate, thereby lowering the reaction overpotential and increasing the surface coverage of adsorbed CO species, which promotes subsequent C‐C coupling reactions [70, 73]. Further, organic anions predominantly affect CO_2_RR through catalyst surface modification and restructuring. For example, propionate (C_3_H_5_CO_2_^−^) significantly enhances CO selectivity on the electrode surface, achieving a Faradaic efficiency of 98.7% at −0.8 V versus RHE, substantially higher than that obtained in bicarbonate‐based electrolytes [74]. In a comprehensive study, Ge et al. explored the influence of various anionic surfactants, namely sodium dodecylbenzene sulfonate, sodium lauryl sulfate, sodium monolauryl phosphate, and sodium laurate, on CO_2_ electroreduction in bicarbonate electrolytes. The incorporation of these surfactants substantially enhanced CO selectivity, yielding CO Faradaic efficiencies of 89.7%, 97.5%, 98.4%, and 98.9%, respectively, at −1.2 V versus RHE, compared with only 53.1% in the absence of surfactants [75]. Dodecyl phosphate has been employed to enhance CO_2_ electroreduction by reorganizing the hydrogen‐bonding network at the electrode‐electrolyte interface, increasing the fraction of free water and thereby boosting CO Faradaic efficiency from 70% to 98% at −1.0 V versus RHE while maintaining over 90% selectivity during prolonged flow‐cell operation [76].

#### 3.1.3 Mass Transport Dynamics

Mass transport plays a pivotal role in determining the surface concentrations of reactants and intermediates during CO_2_ reduction, as reaction kinetics are highly sensitive to local CO_2_ availability, interfacial pH, and applied overpotential. Consequently, the formation rates of individual products can be quantitatively described by Tafel‐type kinetic relationships based on these local reaction conditions as follows in Equation (1):

(1) $$\begin{array}{cc} i_{k} & =-i_{o,k}\;\left(C_{\mathrm{CO}2}/C_{\mathrm{ref}}\right)\;\gamma_{\mathrm{CO}2,k}\;\exp\left(-\gamma_{\mathrm{pH},K,\mathrm{SHE}}\mathrm{pH}\right)\;\\ & \;\exp\left(-\alpha_{c,k}F\eta_{k}/\mathrm{RT}\right) \end{array}$$

In this expression, i_o,k_ denotes the exchange current density, ^γ^_CO2,k_ represents the reaction order with respect to CO_2_ concentration, γ_pH,K_ describes the dependence of current density on pH, and α_c,k_ and η_k_ correspond to the charge‐transfer coefficient and overpotential associated with product k.

The Tafel rate expression assumes a single rate‐determining step, while all preceding and subsequent elementary steps remain in quasi‐equilibrium. Accordingly, charge‐transfer kinetics are represented using semi‐empirical lumped parameters that characterize the rate‐limiting electrochemical process. Although the influence of the electrical double layer is implicitly incorporated into kinetic parameters such as the charge‐transfer coefficient and exchange current density, the local CO_2_ concentration and interfacial pH remain critical determinants of CO_2_ reduction performance. Variations in these local reaction conditions directly affect the formation rates and selectivity of the individual CO_2_RR products. This insight underscores the importance of engineering the interfacial chemical microenvironment to regulate local reaction conditions, thereby enhancing overall current densities and steering product selectivity toward desired CO_2_ reduction products [77].

#### 3.1.4 Interfacial Field Effect

Microenvironment engineering strategies utilize dynamically applied external fields to actively regulate interfacial reaction conditions, thereby enabling real‐time optimization of electrochemical reaction kinetics and product selectivity. Pulsed electrolysis dynamically regulates the interfacial microenvironment through periodic potential modulation, wherein cathodic relaxation periods facilitate CO_2_ replenishment at the electrode surface and promote the redistribution of interfacial ions, while anodic pulses reduce *H surface coverage, thereby suppressing the competing HER. On Ag catalysts, appropriately applied anodic potentials promote the adsorption of monodentate formate (*OCHO_M_) intermediates, thereby facilitating the selective conversion of CO_2_ to formate. In contrast, on Cu catalysts, mild oxidative pulses induce the formation of roughened, undercoordinated surface sites, which accelerate the consumption of linearly adsorbed *CO intermediates and consequently enhance C─C coupling toward C_2__+_ products [78]. Operando X‐ray absorption spectroscopy and X‐ray diffraction studies of Cu_2_O nanocubes‐derived catalysts have revealed that precise control of alternating pulse durations maintains an optimal balance between metallic Cu^0^ domains and distorted Cu(I)/Cu(II) oxide species. This dynamically stabilized mixed‐valence surface state promotes CO dimerization and substantially enhances ethanol selectivity [79]. The underlying mechanism of this catalyst restructuring has been revealed through integrated operando spectroscopic and microscopic studies on Cu(100) single‐crystal surfaces, which show that transient anodic pulses drive selective dissolution and subsequent redeposition of Cu atoms, resulting in the formation of metastable truncated pyramidal architectures enriched with high‐index (n10) facets. The enhanced C─C coupling activity and elevated C_2__+_ product selectivity observed under pulsed CO_2_ electroreduction originate from the synergistic interplay between defect‐rich high‐index (n10) surface facets and a stabilized subsurface reservoir of oxygen‐containing Cu(I) species, which together create a highly favorable catalytic environment for multicarbon product formation [80].

The application of an external magnetic field can markedly enhance electrocatalytic CO_2_ reduction through multiple mechanisms, including magnetohydrodynamic effects, spin‐state regulation, and electronic structure modulation. At the macroscopic level, a magnetic field applied perpendicular to the ionic current induces a Lorentz force on migrating charged species, generating magnetohydrodynamic convection that alleviates concentration polarization and minimizes local pH gradients at the electrode‐electrolyte interface [81]. The resulting enhancement in mass transport mitigates diffusion limitations, leading to higher current densities and improved CO_2_RR selectivity relative to HER. The applied magnetic field promotes Δg‐mechanism‐induced intersystem crossing of spin‐correlated [CO_2_^•−^···H^•^] radical pairs from triplet to singlet states. Since the formation of the adsorbed formate (*HCOO^−^) intermediate is spin‐allowed only from singlet radical pairs according to the Pauli exclusion principle, this spin‐state conversion effectively circumvents the spin‐forbidden triplet pathway, thereby substantially enhancing HCOOH production [82]. At the intrinsic electronic level, applying a magnetic field can regulate the magnetic domain structure of ferromagnetic catalysts, such as Ni@NC, inducing a transition from a multidomain to a highly ordered single‐domain state that facilitates electron transfer. This magnetic ordering shifts the metal d‐band center closer to the Fermi level, thereby strengthening *COOH adsorption, lowering the activation energy for CO_2_ reduction, and significantly enhancing catalytic performance, resulting in nearly quantitative CO selectivity and substantially increased current densities [83]. Collectively, these approaches highlight that precise engineering of the physicochemical microenvironment at the electrode‐electrolyte interface is crucial for regulating reaction pathways, thereby enabling highly efficient and selective electrochemical CO_2_ reduction.

### 3.2 Computational Frameworks for Realistic CO_2_RR Electrocatalyst Design

Computational modelling has become a central pillar in the rational design of electrocatalysts for electrochemical CO_2_RR, enabling mechanistic understanding and predictive insights that are often inaccessible experimentally [84]. Early density functional theory (DFT) studies successfully correlated adsorption energetics of key intermediates with catalytic activity and selectivity, leading to the widely adopted classification of metals into CO^−^, HCOOH‐, or hydrocarbon‐selective catalysts [85, 86]. However, it is now well established that static, zero‐charge adsorption energies alone are insufficient to describe CO_2_RR under realistic electrochemical conditions, where electrode potential, interfacial electric fields, electrolyte cations, solvation, surface coverage, and dynamic surface restructuring play pivotal roles.

**FIGURE 4:**
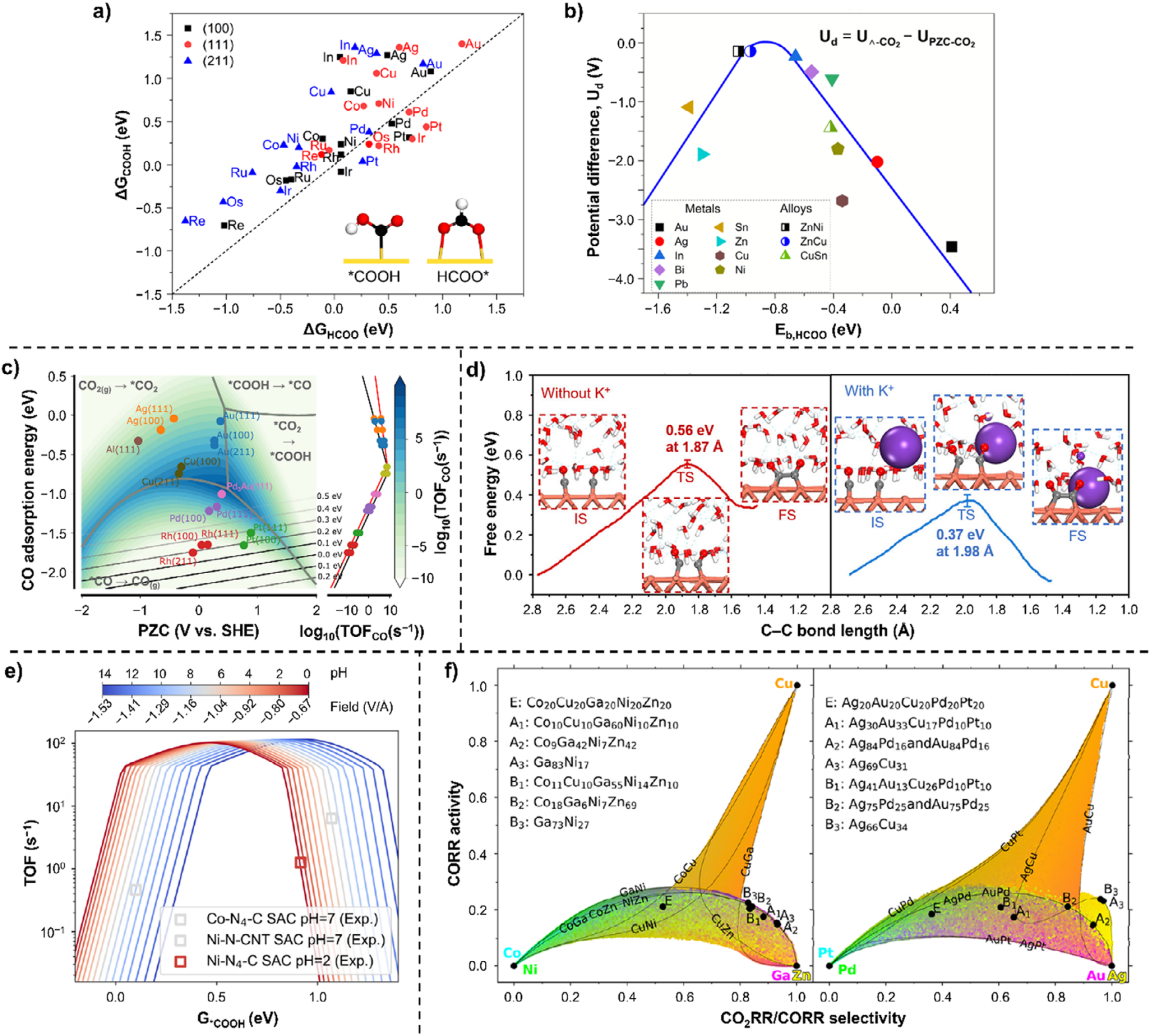
(a) Calculated reaction free energies (ΔG) of HCOO* and *COOH formation on 13 metals. (b) The volcano diagram constructed by the potential difference (U_d_) as a function of HCOO binding energy (E_b_). Reproduced with permission from [87]. Copyright 2024, American Chemical Society. (c) Charge‐corrected descriptor‐based volcano plot for CO_2_‐to‐CO activity at −1 V_SHE_, shown as a function of potential of zero charge (PZC) and CO adsorption energy. Contours represent the predicted turnover frequency (TOF). Reproduced with permission from [88]. Copyrights 2023, Nature Publishing Group. (d) Grand free energy profiles for *CO—*CO coupling on Cu(100) at −1.5 V_SHE_ calculated using the slow‐growth approach, in the absence and presence of K^+^ ions in the electrolyte. Reproduced with permission from [95]. Copyright 2024, American Chemical Society. (e) The pH‐dependent microkinetic volcano model for d‐block M‐N‐C catalysts in CO_2_RR toward CO generation. Reproduced with permission from [100]. Copyright 2025, AIP Publishing. (f) Selectivity‐activity space for CO_2_RR to CO on multicomponent alloys, illustrating the trade‐off between CO_2_RR/CORR selectivity and CORR activity across alloy compositions. Reproduced with permission from [101]. Copyright 2020, American Chemical Society.

A major conceptual advance has been the explicit treatment of electrode potential within grand‐canonical or constant‐potential DFT frameworks. Zhao and Wang [87] revisited two‐electron CO_2_RR on transition metals using grand canonical DFT combined with a hybrid solvent model and demonstrated that product selectivity is governed by the kinetic accessibility of activated CO_2_^−^ states rather than static comparisons of *COOH and HCOO* adsorption energies. Although HCOO* is thermodynamically more stable than *COOH on most metals (Figure 4a), Au remains CO‐selective because formation of the ∧‐shaped CO_2_^−^ intermediate is kinetically inaccessible, suppressing the HCOO pathway. In contrast, In stabilizes the ∧‐CO_2_^−^ intermediate at much lower overpotentials, enabling facile HCOO formation and high HCOOH selectivity. This computed contrast directly reflects the experimentally observed product distributions on these metals, where Au is typically CO‐selective while In‐based catalysts preferentially produce formate/formic acid. This insight led to a new descriptor: the potential difference between the onset of stable ∧‐CO_2_^−^ formation and the potential of zero charge (PZC), which yields a volcano‐type trend across metals and alloys (Figure 4b) and quantitatively rationalizes experimentally observed HCOOH selectivity. More broadly, this work highlights the central role of surface charging and charge transfer in CO_2_RR. These ideas were reinforced by Ringe [88], who showed that adsorption‐energy‐only descriptor spaces fundamentally fail in electrocatalysis because they neglect surface charging and electric double‐layer effects. By explicitly incorporating the PZC alongside the CO adsorption energy, a two‐dimensional descriptor space emerges in which catalysts with identical adsorption energetics exhibit vastly different CO_2_RR activities (Figure 4c), directly reflecting differences in charge transfer to the CO_2_ intermediate. This framework breaks traditional scaling relations and quantitatively reproduces experimentally observed trends in CO_2_RR activity and selectivity. Experiment‐theory agreement has also been demonstrated for Au, where multiscale modelling with explicit double‐layer charging reproduces the measured pH‐invariant CO partial current on the standard hydrogen electrode (SHE) scale and the observed polarization behavior, showing that CO_2_ adsorption and field‐stabilization of *CO_2_ govern the experimentally observed CO selectivity [89]. Likewise, constant‐potential DFT combined with pH‐dependent kinetic measurements on Cu links distinct product channels to distinct experimental signatures: C_2+_ formation remains nearly pH‐independent on the SHE scale, whereas CH_4_ formation exhibits a pronounced pH dependence, directly connecting computed elementary steps to observed product selectivity [90].

Electrolyte cation effects further reinforce the centrality of interfacial electrostatics in CO_2_RR and are now recognized as fundamental determinants of selectivity rather than secondary experimental variables [62, 91, 92, 93]. Qin et al. [94] employed ab initio molecular dynamics (AIMD) to examine how interfacial cation identity and concentration influence CO_2_RR and competing HER at Au‐water interfaces. They demonstrate that increasing the local alkali metal cation (AM) concentration, particularly K^+^, significantly lowers the CO_2_ activation barrier through short‐range K‐O coordination with the *CO_2_ intermediate, thereby facilitating CO_2_RR, which follows the trend 2K^+^ > 1K^+^ > 2Li^+^ > 1Li^+^ > 0AM^+^. In contrast, HER kinetics are mainly governed by interfacial water structure and hydrogen‐bond connectivity, exhibiting an opposite trend. Complementarily, Qin et al. [95] used constant‐potential AIMD simulations to show that K^+^ can undergo specific adsorption in the inner Helmholtz plane on Cu surfaces during *CO—*CO coupling, lowering the C‐C coupling barrier from 0.56 to 0.37 eV on Cu(100), as illustrated in Figure 4d. Electronic structure analysis reveals that this barrier reduction originates from charge redistribution between specifically adsorbed K^+^, *CO, and adjacent Cu sites, thereby stabilizing the transition state and enhancing C_2+_ selectivity. These cation effects are not only theoretical constructs but are also reflected in measured selectivity trends. Combined kinetic experiments and simulations have shown that CO and C_2_H_4_ formation increase systematically with larger alkali cations, whereas CH_4_ follows a different scaling behavior, consistent with cation‐coupled electron transfer for CO/C_2_ pathways but proton‐coupled steps for methane formation [93]. On Au, experiment‐AIMD studies further show that the balance between CO_2_RR and HER depends on how specific cations simultaneously stabilize *CO_2_^−^ and promote or suppress interfacial water dissociation, thereby shifting the experimentally observed CO selectivity across cation identity [96].

Beyond potential effects, surface coverage has emerged as a critical variable controlling CO_2_RR selectivity. For C_1_ products, coverage‐aware DFT studies on several monometallic catalysts have shown that finite populations of *COOH and HCOO* qualitatively alter adsorption energetics and resolve long‐standing discrepancies between theory and experiment. Morrison et al. [97] demonstrated that lateral interactions introduce systematic errors in isolated‐adsorbate models, while coverage‐dependent energy ratios provide robust descriptors for CO versus HCOOH selectivity. Related ideas were generalized by Christensen et al. [98] using data‐driven classification, principal component analysis, and reaction‐pathway analysis. For C_2+_ formation on Cu, coverage effects are even more crucial. Operando Raman spectroscopy by Zhan et al. [99] directly measured potential‐dependent *CO coverage on Cu_2_O nanocubes and revealed a volcano‐type dependence of C_2+_ F.E. on *CO coverage. Complementary DFT analysis showed that only intermediate *CO coverages stabilize favorable C‐C coupling geometries, underscoring that selectivity is governed by ensemble statistics rather than single‐site energetics. This is a particularly strong case where modelling directly correlates with experiment: the spectroscopically measured *CO coverage and the F.E. for C_2+_ products exhibit matching volcano‐type trends, while DFT identifies the corresponding coverage window in which atop/bridge CO ensembles most effectively promote C─C coupling. Thus, coverage‐dependent modelling is required not only for mechanistic completeness but also for reproducing experimentally resolved selectivity trends.

A decisive extension of potential‐ and coverage‐aware modelling to realistic Cu surfaces was provided by Cheng et al. [102] Using grand canonical DFT combined with global optimization of CO adlayers and kinetic modelling, it has been demonstrated that ideal planar Cu(111) and Cu(100) facets are essentially inactive for CO_2_RR due to negligible CO coverage under reaction conditions. Instead, CO_2_RR proceeds on stepped and kinked surfaces formed in situ, with strong CO binding at defect sites acting as the thermodynamic driving force for surface restructuring. Importantly, the catalytically active motifs were identified not as the defect atoms themselves, but as square Cu ensembles adjacent to defects, which synergistically stabilize CO and promote C‐C coupling. This work fundamentally revises facet‐based interpretations of Cu selectivity and establishes dynamic restructuring as an intrinsic component of CO_2_RR catalysis. Related strategies to manipulate *CO binding and coverage have emerged in confined and heterogeneous nanointerfaces. Zhang et al. [103] showed that alloying Cu with Zn creates neighboring binary sites with asymmetric CO binding energies. DFT‐derived global reaction phase diagrams and microkinetic modelling revealed that such asymmetry lowers C─C coupling barriers beyond the limits of scaling relations, while operando experiments confirmed enhanced *CO coverage and C_2+_ selectivity even under acidic conditions.

Recent data‐driven studies have begun to extend potential‐aware modelling to explicitly capture pH effects. Chu et al. [100] analyzed CO_2_‐to‐CO activity on d‐block transition metal‐based SACs by combining large‐scale experimental data mining with pH‐ and electric‐field‐coupled microkinetic modelling. Screening 101 M‐N‐C SACs against 939 experimental data points, they demonstrated that CO selectivity depends sensitively on pH‐dependent interfacial electric fields rather than adsorption energetics alone. This behavior is captured by pH‐dependent microkinetic volcano plots (Figure 4e), which reproduce experimental turnover frequencies on the reversible hydrogen electrode scale.

As CO_2_RR modelling increasingly incorporates potential dependence, coverage, restructuring, and ensemble effects, the resulting combinatorial complexity has driven the adoption of machine learning (ML) as a key enabling tool. Zhang et al. [104] reviewed how ML models, from kernel methods to graph neural networks (GNNs), can act as fast surrogates for DFT‐level energetics, enabling high‐throughput screening of complex materials such as HEAs and SACs. Pedersen et al. present one of the earliest and clearest examples in CO_2_RR, using DFT and supervised ML to predict *CO and *H adsorption energies on all local surface sites of two HEAs, CoCuGaNiZn and AgAuCuPdPt, on the (111) facet [101]. By training ML models to map local atomic environments to adsorption energies, they can efficiently evaluate the distribution of site‑specific descriptor values as a function of alloy composition. This enables optimization of compositions that statistically favor sites with weak *H binding (suppressing HER) and strong *CO binding (promoting deeper reduction of CO_2_/CO). This active‐motif, ensemble‐based design principle is reflected both in selectivity‐activity maps that delineate the accessible catalytic performance space (Figure 4f) and in adsorption‐energy distributions that show how composition reshapes the populations of catalytically relevant surface sites. Mok et al. [105] generalized this approach by integrating motif‐based ML predictions with potential‐dependent selectivity maps, screening over two million motifs across 465 binary alloys and discovering previously unreported and promising behavior of Cu‐Ga and Cu‐Pd catalysts. Inverse design strategies further extend ML‐assisted discovery. Song et al. [106] combined generative models with supervised GNNs to directly generate alloy surfaces optimized for CO_2_RR‐relevant CO adsorption energies. Complementary to such inverse design approaches, Sun and Huang developed a first‐principles ML framework for single‐atom and atomic catalysts on carbon supports, trained on more than 15,000 DFT data points [107]. Their analysis revealed site‐family‐specific scaling relationships for hydrogenation steps and introduced a pH‐aware calibration strategy that aligns ML‐predicted energetics with experimentally relevant potential scales.

Beyond DFT‐derived screening, recent studies have increasingly demonstrated the direct integration of experiment‐ML for predicting and optimizing CO_2_RR selectivity. Guo et al. [108] established an iterative workflow combining catalyst synthesis, electrochemical testing, ML analysis, and catalyst redesign, ultimately identifying Cu‐based catalysts that selectively produce CO, HCOOH, and C_2+_ products. Similarly, Setyowati et al. [109] trained ML models on experimental Ag CO_2_RR datasets to predict electrolysis conditions that maximize CO production and achieve targeted H_2_/CO syngas ratios, with the proposed optimal conditions subsequently validated experimentally. More recently, Yao et al. [110] developed ML models trained on 378 experimental datasets for Cu and Cu‐derived catalysts that accurately predict product‐specific F.E. and identify applied potential and catalyst morphology as the dominant factors governing selectivity.

In summary, computational CO_2_RR research is transitioning from static, descriptor‐based interpretations toward dynamic, multiscale, and data‐integrated modelling paradigms. Continued progress will depend not on refining a single method, but on coherently integrating constant‐potential DFT, coverage and ensemble theory, restructuring dynamics, mechanistic fidelity, and machine learning. Achieving this integration represents both the grand challenge and the greatest opportunity for computationally guided CO_2_RR catalyst design.

## 4 Design of Electrocatalytic Material Product Selectivity

### 4.1 Copper Catalysts: Selectivity vs. Instability

**FIGURE 5:**
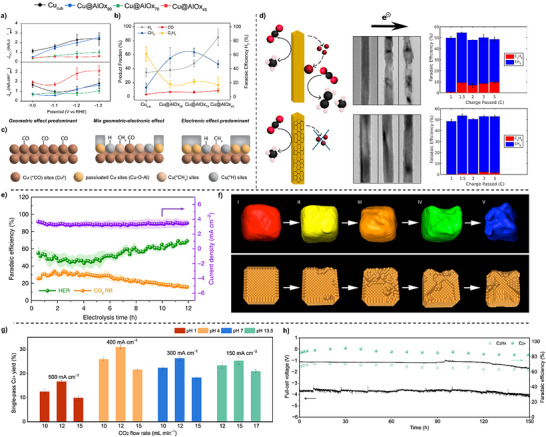
CO_2_RR performance as a probe of the interplay between geometric and electronic effects with varying coating porosity. (a) Geometric partial current densities for CO_2_ (J_CO2_) and H_2_ (J_H2_) for Cu NCs (black), Cu@AlOx_90_ (blue), Cu@AlOx_70_ (green), and Cu@AlOx_45_ (red) as a function of applied potential. (b) CO_2_RR product distribution at −1.2 V vs. RHE for the different catalysts, showing the fraction of H_2_). (c) Schematic illustration of active sites influenced by electronic effects after oxide coating. Reproduced with permission [112]. Copyright 2025, American Chemical Society. (d) Schematic illustrating the correlation between morphology (TEM) and product selectivity for Cu NWs and rGO‐wrapped Cu NWs. Reproduced with permission [113]. Copyright 2017, American Chemical Society. (e) Temporal evolution of FE and current density for 41 nm CuNCs. (f) 3D electron tomography images of a representative Cu_cub_ acquired at various points during 12 h of CO_2_RR (0.1 M KHCO_3_ electrolyte, chronoamperometry at −1.1 V vs. RHE). Reproduced with permission [114]. Copyright 2018, Nature Publishing Group. (g) Single‐pass C_2+_ yields of nanoporous Cu_0.9_Zn_0.1_ measured at varying CO_2_ flow rates and current densities across pH values of 1, 4, 7, and 13.5 in a 13.5 cm^2^ electrochemical cell. (h) CO_2_ reduction stability of graphite/carbon nanoparticle‐coated Cu_0.9_Zn_0.1_ evaluated at 150 mA cm^−^^2^ in a slim two‐electrode flow cell, showing FE for C_2+_ products and ethylene. Reproduced with permission [103]. Copyright 2023, Nature Publishing Group.

Copper remains a central and unavoidable benchmark in CO_2_RR catalysis [111], owing to its optimal binding energies for key intermediates that facilitate efficient CO_2_ reduction, resulting in up to 14 reduced products under neutral conditions. The wide range of product distribution depends on multiple factors, including the method of catalyst preparation. Given that single crystals and shape‐controlled nanocrystals (NCs) are widely recognized for their facet‐specific catalytic behavior, the structure‐dependent selectivity of Cu becomes particularly significant, necessitating precise integration with electrode architectures. Cubic Cu NCs (Cu_cub_ NCs), which predominantly expose (100) facets, provide an ideal platform for translating insights from single‐crystal studies to more applied systems and a deeper understanding of reaction pathways during CO_2_RR. Buonsanti's group leveraged the tunable porosity of coatings to systematically modulate the balance between geometric and electronic effects at Cu(100) active sites, enabling correlation of active‐site characteristics with their sensitivity to microenvironmental effects during CO_2_RR. They synthesized well‐defined Cu_cub_ NCs by the colloidal method and a series of Cu_cub_ encapsulated by organic/inorganic hybrid alumina coating of varying porosity. While an alumina coating suppresses structural reconstruction in Cu NCs, the presence of organic ligands, such as oleic acid, imparts porosity to the coating, thereby preserving accessibility of the Cu surface. Based on the porosity results, the samples were designated as Cu@AlOx_90_, Cu@AlOx_70_, and Cu@AlOx_45_. Figure 5a,b presents trends in selective product formation with different catalysts. On bare Cu_cub_, the geometric effects of (100) facet dominate, promoting strong ^∗^CO adsorption and favoring ethylene formation via C‐C coupling. However, the geometric CO_2_ partial current density decreases monotonically with increasing porosity, consistent with a reduction in the number of active CO_2_RR sites as copper becomes passivated by the oxide layer. At the same time, the geometric H_2_ partial current density increases with increasing porosity, suggesting that a fraction of the remaining unpassivated Cu sites shifts from CO_2_ reduction to HER upon coating. While ethylene is the dominant CO_2_RR product on bare Cu_cub_ NCs, as reported previously, alumina coating shifts selectivity toward methane, which becomes the predominant product (>50%) in all coated samples. Also, the structural integrity of Cu_cub_ is largely intact upon coating with AlO_x_. Next, the electrochemically active surface area (ECSA) was measured to reflect the intrinsic activity of CO_2_RR‐active catalytic sites, and the trend was observed as Cu@AlOx_90_> Cu@AlOx_70_> Cu NCs > Cu@AlOx_45_. This means that, even though the total number of CO_2_RR active sites gradually decreased with AlO_x_ coating, the intrinsic activity of Cu@AlOx_90_ and Cu@AlOx_70_ was found to be higher than that of uncoated Cu_cub_. After accounting for mass transport, spatial constraints on C‐C coupling, pH effects, and alumina‐intermediate interactions, they concluded that the selective formation of ethylene is a geometric effect of the (100) facet (Figure 5c). Consequently, shifts in product distribution serve as an indirect probe of the relative contributions of geometric versus electronic effects to reactivity. The selectivity changes induced by coating are predominantly electronic in origin. Furthermore, noting that Cs^+^ promotes C_2+_ products, they replaced KHCO_3_ with CsHCO_3_ and observed a pronounced increase in ethylene production across all catalysts. Thus, using this platform, they propose that catalytically active sites governed by electronic effects are more sensitive to changes in the local microenvironment, as exemplified by alkali cations in the electrolyte [112].

To understand the selectivity trends for CO_2_RR on Cu surface, Yang and co‐workers investigated the electrocatalytic performance of ultrathin five‐fold‐twinned Cu nanowires (NWs). These Cu NW catalysts exhibited high selectivity toward CH_4_, achieving a F.E. of 55% at −1.25 V versus the reversible hydrogen electrode, while all other carbon products showed F.E. below 5%. Figure 5d shows the correlation of morphology and selectivity. The mechanistic model of CO_2_RR, TEM images and product selectivity over bare Cu NWs and Cu NW wrapped with reduced graphene oxide (rGO) show that the product distribution strongly depends on the structural stability of the nanowires during operation. Morphological evolution under electrolysis altered selectivity, whereas encapsulation with rGO effectively stabilized the nanowire architecture and maintained the intrinsic catalytic selectivity. These findings indicate that the product selectivity of Cu nanowires is strongly governed by their morphology, and that hydrocarbon formation can be tuned by either inducing or suppressing structural transformations [113].

Again, to understand and elucidate the catalyst degradation mechanism, Huang et al. synthesized three different‐sized, monodisperse Cu_cub_ particles via colloidal synthesis and monitored their structural and catalytic behavior. The study identified potential‐driven fragmentation as the primary degradation mechanism, leading to the formation of Cu nanoclusters with sizes of approximately 2–4 nm. Catalyst stability was assessed by interrupting the electrolysis at predetermined intervals and recovering the Cu_cub_ for microscopic characterization. Structural analysis revealed a progressive breakdown of the individual nano‐cubes during operation, followed by aggregation and eventual coalescence at extended reaction times. After 12 h, the Cu_cub_ are fused into aggregated structures, leaving no intact individual nano‐cubes. To assess whether these structural changes influence catalytic performance, the electrocatalytic behavior was monitored during prolonged electrolysis. As shown in Figure 5e, the FE for CO_2_ reduction declines modestly after 6 h, accompanied by an increase in hydrogen evolution, while the overall current density remains essentially constant over 12 h due to the offsetting nature of these two trends. The 3D electron tomography (Figure 5f) reveals that the Cu_cub_ undergo a pitting process, resulting in the formation of nanoscale pinholes along the cube edges. This observation aligns with theoretical predictions identifying the edge‐facet interface as the most favorable site for C─C coupling [114].

During C‐C coupling in CO_2_R, the rate‐determining step to the C_2+_ product, efficiency usually remains low, and product stability is poor. To overcome this bottleneck, Zhang et al. developed an alloying strategy that creates neighboring binary active sites with asymmetric CO binding energies, enabling CO_2_‐to‐C_2+_ electroreduction beyond the activity limits imposed by scaling relations on monometallic surfaces. They experimentally synthesized a series of Zn‐incorporated Cu catalysts that exhibit enhanced asymmetric *CO binding and elevated surface *CO coverage, accelerating C‐C coupling and subsequent hydrogenation during electrochemical CO_2_ reduction. Strategic optimization of the nanointerface reaction environment further suppresses hydrogen evolution while boosting CO2 utilization under acidic conditions. This nanoporous architecture creates a favorable local microenvironment that concentrates ions and intermediates at the Cu_0.9_Zn_0.1_/electrolyte/CO_2_ triple interface, thereby enhancing C_2+_ conversion. They attained a single‐pass C_2+_ yield of 31 ± 2% in pH 4 electrolyte at 400 mA cm^−2^ with a CO_2_ flow rate of 12 mL min^−^^1^ in a 13.5 cm^2^ cell (Figure 5g). This represents a twofold improvement over the previous benchmark of 16.5% achieved with fluorine‐modified Cu in alkaline electrolytes. Finally, they evaluated CO_2_RR stability and full‐cell energy efficiency in a two‐electrode electrolyzer with alternating current cycles (−150 mA cm^−2^ for 120 s, −1 mA cm^−2^ for 30 s). They achieved stable C_2+_ and ethylene production with FE of ∼90% and ∼70%, respectively, at 150 mA cm^−2^ for over 150 h (Figure 5h) [103]. Nonetheless, the structure of copper‐based electrodes is not static during operation, as they experience reconstruction phenomena commonly encountered in heterogeneous catalytic systems, which will be examined in detail in the following section.

CO_2_ pressure is a critical parameter governing the selectivity of the electrochemical CO2 reduction reaction over copper catalysts by altering the surface concentrations of key reaction intermediates, modulating the local proton environment, and suppressing HER, thereby directing product formation toward specific reduction pathways. The substantial increase in dissolved CO_2_ concentration under elevated pressure restructures the interfacial reaction environment, favoring CO_2_ adsorption and activation over proton‐reduction pathways, thereby effectively suppressing the HER [115]. Under high‐pressure conditions (50‐60 bar), increased CO_2_ availability at the catalyst interface enhances the coverage of reactive carbonaceous species, shifting the reaction network toward carbon hydrogenation routes and selectively generating formate as the dominant product. Such pressure‐induced selectivity has enabled tailored Cu‐based catalysts to deliver formate Faradaic efficiencies of up to 98% [116]. Conversely, optimal selectivity toward multi‐carbon products, such as ethylene, is typically achieved at moderate CO_2_ partial pressures, which facilitate the surface processes required for C‐C bond formation [115, 117]. Limiting interfacial CO_2_ availability enhances the accessibility of active sites necessary for *CO coupling and induces a more alkaline microenvironment, synergistically favoring the formation of C_2__+_ products [118]. Strategic modulation of CO_2_ pressure in conjunction with engineered catalyst architectures, including asymmetric Cu‐N_2_ single‐atom sites, can effectively accelerate kinetically sluggish reduction steps and steer CO_2_RR toward highly selective methane production. The synergistic effect of enhanced CO_2_ availability at elevated pressures and the strong adsorption affinity of engineered active sites for *CO promotes sustained intermediate retention, thereby favoring stepwise hydrogenation pathways leading to more deeply reduced products [119].

Hence, Cu remains the benchmark catalyst for CO_2_RR because its intermediate CO adsorption strength is well balanced: it binds CO strongly enough to favor further reduction rather than desorption to form CO and formic acid, while its relatively weak H adsorption helps suppress the HER. This unique combination enables Cu to produce a broad spectrum of reduced products, including both C_1_ and C_2+_ species. Thus, engineered surfaces that sustain high CO coverage, promote CO‐CO (or CO‐CHO) coupling on moderately binding sites, and prevent overly strong H binding. The activity and selectivity of Cu electrocatalysts are highly sensitive to structure, morphology, and local microenvironment: Cu(100)‐rich cubic NCs favor ethylene formation, while AlO_x_ coatings, porosity, and alkali cations can shift selectivity and alter the balance between CO_2_RR and HER. In parallel, Cu nanowires and cubic NCs undergo dynamic reconstruction, fragmentation, pitting, and coalescence, which strongly affect stability and product distribution. More recent alloying strategies, such as Zn‐incorporated Cu, demonstrate that engineering asymmetric active sites and nanoporous interfaces can suppress HER, enhance CO_2_ utilization, and improve C_2+_ selectivity and durability. Overall, these results highlight that effective CO_2_RR on Cu depends not only on intrinsic surface chemistry but also on controlled architecture, interfacial design, and resistance to electrochemical reconstruction.

### 4.2 Understanding the Intermediates through Operando Techniques

**FIGURE 6:**
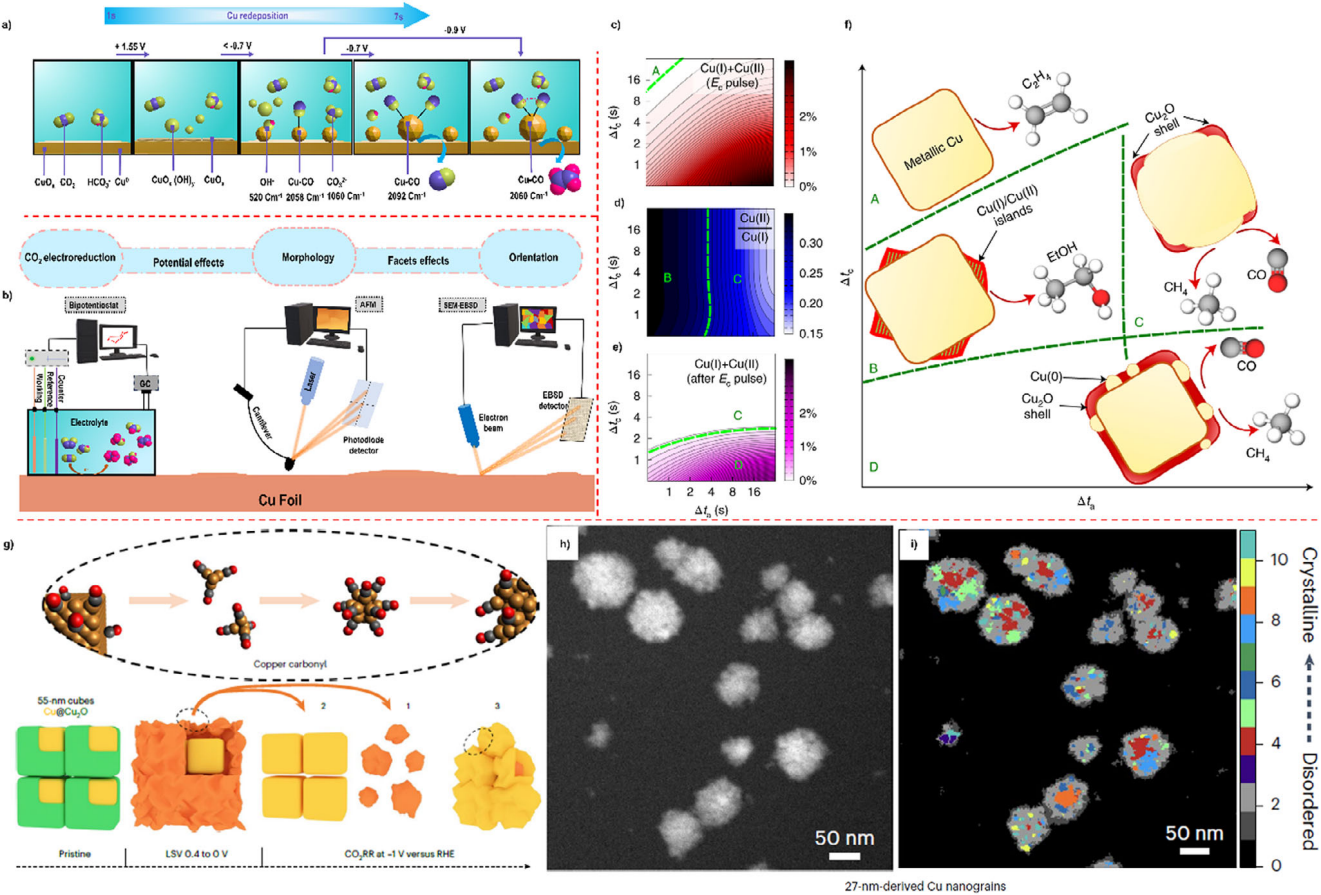
(a) Illustration of the dynamic processes taking place on Cu‐MP under CO_2_RR conditions, monitored by in situ time‐resolved SERS. Reproduced with permission [120]. Copyright 2021, Wiley‐VCH. (b) Schematic depiction of the developed analytical framework, enabling the investigation of potential‐dependent facet restructuring under CO_2_ electroreduction conditions using identical‐grain AFM and EBSD. Reproduced with permission [121]. Copyright 2025, Wiley‐VCH. Effect of anodic (Δt_a_) and cathodic (Δt_c_) pulse durations. (c–e) XAS analysis of Cu nanocubes under E_a_ = 0.6 V, showing average oxide concentration during the cathodic pulse, Cu(II)/Cu(I) ratio, and residual oxide after each nth cathodic pulse. (f) Schematic illustration of catalyst structure and composition during a cathodic pulse derived from XAS and XRD. Reproduced with permission [79]. Copyright 2022, Nature Publishing Group. (g) Schematic illustration of carbonyl‐induced restructuring in 55‐nm Cu@Cu_2_O nanocubes. (h, i) Operando STEM imaging and 4D‐STEM clustering analysis of nanograins formed from 27‐nm Cu nanocubes. Reproduced with permission [122]. Copyright 2025, Nature Publishing Group.

Surface reconstruction during heterogeneous electrocatalysis is almost inevitable, necessitating an atomistic/molecular insight into how they evolve and (de)activate under electrochemical conditions [123]. Such reconstructions are mostly advantageous, as they promote the formation of additional active sites; however, they may also exert detrimental effects. During reconstruction events, the formation and coupling of reactive intermediates mostly occur at sub‐second timescales, requiring operando, time‐resolved spectroscopic techniques capable of capturing dynamic surface evolution and adsorption‐desorption processes in real time to unveil the mechanistic pathway [124]. Raman and IR spectroscopy are particularly powerful operando techniques for identifying adsorbed intermediates and tracking surface species in real time, thereby providing critical insights into electrocatalytic reaction mechanisms; [125] however, their effectiveness can be limited by weak signal intensities, overlapping vibrational bands, and interference from the electrochemical environment, which may complicate the accurate identification of reaction intermediates. In such cases, theoretical calculations are indispensable because computed vibrational frequencies, Stark shifts, adsorption energetics, and coverage‐dependent spectral responses can help distinguish among adsorbed CO, carbonate, hydroxide, and C─C coupled intermediates, thereby resolving assignments that cannot be unambiguously established from experiment alone [126, 127]. Weckhuysen and co‐workers employed time‐resolved surface‐enhanced Raman spectroscopy with sub‐second temporal resolution to investigate the steady‐state surface adsorption of CO species and to probe the surface evolution of Cu electrodes during electrochemical CO_2_ reduction (Figure 6a) [120]. To overcome the inherently low Raman scattering cross sections, which typically demand minute‐long acquisition times to achieve adequate signal‐to‐noise ratios and thereby obscure rapid kinetic events, they applied a targeted electrochemical anodic pretreatment to a mechanically polished polycrystalline Cu (Cu‐MP) electrode, generating SERS‐active “hotspots”. During anodic polarization at 1.55 V for 120 s, the Cu surface undergoes partial dissolution, releasing Cu^2+^ and CuO_x_ (OH)_y_ species into the interfacial electrolyte [128]. Subsequent switching to a reducing potential (−0.4 V) triggers rapid redeposition and nanoparticle growth (50–150 nm), as confirmed by ex‐situ AFM [129, 130], leading to a pronounced increase in surface roughness (RMS 13 to 41 nm) and an eight‐fold enhancement of the double‐layer capacitance. Moreover, at a cathodic bias of ‐0.4 V, the anodically formed CuO_x_ layer is rapidly reduced via a proton‐consuming process that occurs concurrently with the hydrogen evolution reaction, inducing local proton depletion and a sharp increase in interfacial pH, which shifts bicarbonate (HCO_3_^−^) to carbonate (CO_3_^2−^). The emergence of a surface‐adsorbed carbonate band at 1060 cm^−1^, which is absent from the bulk electrolyte spectrum dominated by bicarbonate at 1012 cm^−1^, provides direct spectroscopic evidence for the presence of nanostructured SERS‐active “hotspots”. Time‐resolved SERS at a cathodic bias of −0.7 V reveals that early surface reconstruction and rapid local alkalization suppress C‐C coupling within the first ∼7 s of electrolysis. An initially dynamic terrace/step‐edge low‐frequency CO band (LFB‐CO) (∼2058 cm^−1^) evolves, [131] upon completion of Cu redeposition and proton depletion by HER (indicated by the Cu‐OH mode at ∼520 cm^−1^), into an irreversibly formed, static high‐frequency CO band (HFB‐CO) (∼2092 cm^−1^) associated with isolated defect sites, [132] leading to CO trapping and desorption rather than dimerization and thereby favoring monomeric CO release over C_2_ product formation at low overpotential. Additionally, bridged CO serves as a negative spectroscopic control, as its characteristic vibrations appear at subsequently lower wavenumbers (∼2020 or ∼2030 cm^−1^) than the catalytically relevant band at 2040–2060 cm^−1^, thereby excluding multi‐site bridging and enabling unambiguous assignment of ethylene formation to linearly adsorbed CO on step‐edge sites. At ‐0.8 V, the system exhibits behavior like that observed at −0.7 V, whereas at ‐0.9 V the spectrum becomes dominated by a strongly red‐shifted LFB‐CO attributable to the electrochemical Stark effect [133] and CO bond weakening, accompanied by rapid oscillations within 2040–2060 cm^−1^ that persist for 20 min. This dynamic LFB‐CO is attributed to CO adsorbed on step‐edge sites, where elevated overpotential and local geometry promote proximity‐driven C─C coupling over desorption, a mechanism corroborated by a four‐fold increase in ethylene F.E. Theoretical simulations can test whether the observed bands correspond to atop, bridge, or defect‐bound CO, quantify the effects of local electric field and CO coverage on band position and intensity, and discriminate catalytically relevant intermediates from spectator species [99, 126]. Combined operando Raman and DFT studies have shown that potential‐dependent Raman descriptors can be directly related to CO surface coverage and to the preferred C─C coupling configuration, thereby linking spectral evolution with multicarbon selectivity at the atomistic level [99]. More recent work further demonstrated that the combination of in situ SERS and DFT can identify the key intermediates and specific Cu active sites responsible for ethylene and ethanol formation, clarifying mechanistic branch points that spectroscopy alone could not fully resolve [127]. Most studies on the surface reconstruction of Cu catalysts have examined perfect single‐crystal surfaces, such as Cu(001) or Cu(111). To address this knowledge gap, Stam and Weckhuysen's groups systematically elucidated facet‐dependent restructuring of polycrystalline Cu electrodes using electron backscatter diffraction (EBSD) with identical‐grain atomic force microscopy (AFM). Figure 6b presents a schematic representation of the analytic method. While EBSD is an SEM‐based method that enables crystallographic analysis by resolving local crystal orientation, structure, phase composition, and strain at micro‐ to nanoscale dimensions, AFM enables high‐resolution imaging of the 3D surface topography at the catalytic reaction interface. They chose a set of five Cu crystal facets, (001) and (111) as planar, and (114), (212), and (124) as atom‐stepped geometries and examined the statistical slope distribution function to investigate and quantify the asymmetry of surface restructuring across the five Cu facets. Distinct morphologies emerge on the planar facets: Cu (001) develops a square‐shaped morphology after CO_2_RR, reflecting a four‐fold restructuring asymmetry, whereas Cu (111) exhibits a three‐fold asymmetry, giving rise to triangular surface features after CO_2_RR. The (114), (212), and (124) show 2‐fold surface restructuring, leading to morphologies oriented along a single direction after CO_2_RR. They concluded that the post‐CO_2_RR of Cu surface structure retains the asymmetry of its parent crystal facet. Using an electrochemical oxidation‐reduction pulse protocol to probe dynamic surface restructuring and its impact on catalyst stability and performance, they observed four‐, three‐, and two‐fold asymmetries on Cu(001), Cu(111), and stepped facets, respectively, consistent with the surface morphologies observed after CO_2_RR [121]. Although EBSD and AFM provide detailed insight into the orientation‐dependent evolution of surface morphology, they cannot, on their own, determine which reconstructed motifs are thermodynamically stable, kinetically accessible, or active for C─C coupling. In this regard, theoretical modelling plays a central role, as atomistic simulations can connect observed facet‐dependent restructuring with realistic active‐site motifs, including undercoordinated square‐like sites, grain‐boundary structures, and adsorbate‐induced roughened domains formed under reaction conditions [102, 134].

X‐ray absorption spectroscopy (XAS) is well suited for tracking oxidation‐state variations and local coordination environments, thereby providing crucial insights into the dynamic evolution of catalytically active sites during electrochemical operation; however, because XAS predominantly provides ensemble‐averaged information, it may overlook structural and electronic heterogeneities at individual active sites, potentially limiting a complete understanding of catalyst behavior under realistic reaction conditions. Using operando XAS and X‐ray diffraction with sub‐second temporal resolution, Cuenya and Co‐workers revealed that catalysts undergo unexpected, intricate structural and chemical transformations under dynamically changing reaction conditions. They demonstrated that applying a pulsed electrochemical protocol with alternating working and oxidiszing potential intervals can strongly modulate catalysts derived from Cu_2_O nanocubes, enabling the separation of the individual influences of coexisting Cu species on product distribution. Since the oxidation and reduction rates are independent of pulse duration, they estimated them from XAS measurements obtained during pulsed CO_2_ reduction with the longest applied periods (Δt_a_ and Δt_c_ = 30 s). Using these values, they developed a simple kinetic model to describe how the concentrations of Cu(0), Cu(I), and Cu(II) evolve over time for any pulse conditions. This model allows us to examine three key parameters: (1) the average amount of Cu_x_O species during the cathodic pulse, (2) the Cu(II)/Cu(I) ratio, and (3) the residual oxide content after the pulse (Figure 6c–e). Integrating these findings with the catalytic selectivity data reveals four distinct regimes (A‐D, Figure 6f), each characterized by specific surface compositions. Notably, the increased ethanol selectivity observed at low Δt_a_ and intermediate Δt_c_ (region B) arises from an optimal mixture of oxidized and reduced copper species and the presence of a slightly distorted Cu_x_O phase [79]. The interpretation of such ensemble‐averaged XAS and XRD observables requires theoretical modelling, as changes in oxidation state and coordination number alone do not uniquely identify the catalytically relevant surface motifs. Theoretical analysis is therefore essential for translating these averaged signatures into realistic atomistic structures, including mixed Cu(0)/Cu(I) ensembles, strained nanograins, residual‐oxygen configurations, and dynamically restructured oxide‐derived surfaces [135, 136, 137]. In particular, combined operando XAS and theoretical studies have been used to address the long‐standing debate over whether metallic Cu, Cu(I), or mixed‐valence ensembles are responsible for selective C_2_ formation. Large‐scale molecular dynamics simulations and neural network potentials have further clarified the stability and lifetime of trapped oxygen species in oxide‐derived Cu under reaction conditions [135, 137].

While operando X‐ray absorption and scattering techniques yield ensemble‐averaged information, in situ electron microscopy enables direct visualization of morphological, structural, and compositional changes during reconstruction events, both at the nanoscale and atomic levels. Most recently, Yang and co‐workers synthesized (100)‐oriented Cu_cub_ as a model catalyst to uncover the fundamental mechanisms driving dynamic structural reconstruction of 55‐ and 27‐nm Cu_cub_ during CO_2_RR. They used electrochemical liquid‐cell scanning transmission electron microscopy (EC‐STEM) and synchrotron‐based X‐ray spectroscopy under operando conditions to demonstrate that, under CO_2_ reduction conditions, (100)‐oriented Cu@Cu_2_O nanocubes to track the morphological evolution under dynamic conditions (schematic shown in Figure 6g). Operando EC‐STEM studies found that the 55 nm nanocubes formed a spongy shell along their perimeters, likely due to electroreduction of the pristine Cu_2_O shell. In contrast, the 27 nm nanocubes developed a similar but incomplete spongy shell, possibly owing to the higher reactivity of their Cu_2_O shell. To analyze the complex structure of polycrystalline Cu nanograins under CO_2_RR conditions, they employed an operando electrochemical 4D‐STEM diffraction imaging method. The 4D‐STEM technique acquires 2D electron diffraction patterns at every point within a 2D probe‐position grid. Although Cu nanograins derived from 55 and 27 nm Cu_cub_ exhibit similar polycrystalline characteristics, they show markedly different distributions of crystalline and amorphous/disordered domains. The results are presented as false‐color 4D‐STEM maps, in which distinct colors indicate varying degrees of crystallinity in the Cu domains. Colors are indexed from 0 to 11, with 0 (black) corresponding to the SiN_x_/liquid background, 1 (grey) to amorphous or disordered Cu, and 2–11 to crystalline Cu. Nanograins derived from 55 nm nanocubes predominantly contain polycrystalline Cu domains. In contrast, most nanograins derived from 27 nm Cu_cub_ are nearly amorphous or disordered, with only a small fraction being crystalline (Figure 6h,i). 4D‐STEM analysis revealed that the spongy Cu shell is predominantly an amorphous layer surrounding the remaining crystalline Cu_cub_. Using a probe size of approximately 1 nm, nanograin boundaries with distinct crystallographic orientations were resolved within the crystalline domains derived from 55 nm Cu_cub_, as well as between crystalline and amorphous domains derived from 27 nm Cu_cub_. Nanograins derived from 27 nm Cu_cub_ exhibit a greater fraction of amorphous or disordered domains than those derived from 55 nm Cu_cub_, which are predominantly crystalline. This aligns with the more rapid and disordered structural evolution of the smaller, more reactive 27 nm cubes. This further reaffirms the size‐ and potential‐dependent transformation of Cu_cub_ into polycrystalline Cu nanograins while maintaining a high selectivity for C_2_H_4_ and achieving steady‐state performance over an extended period [122]. However, we must note that such characterizations are typically ex situ or constrained by beam sensitivity, limited field of view, and the challenge of capturing true operando dynamics. For this reason, grand‐canonical DFT, molecular dynamics, and kinetic Monte Carlo simulations are particularly valuable, because they can identify metastable CO/H‐stabilized roughened Cu motifs, explain adsorbate‐driven surface lifting and migration, and rationalize why dynamically reconstructed surfaces persist under reaction conditions rather than relaxing to equilibrium structures [102, 138]. Such calculations therefore provide the atomistic and energetic framework needed to interpret operando STEM and 4D‐STEM observations in mechanistic terms.

Thus, to fully unravel electrocatalytic CO_2_ reduction, it is necessary to critically evaluate Raman, IR, XAS, STEM, and 4D‐STEM, since each technique offers unique mechanistic insight while also presenting specific limitations in operando detection, structural resolution, and quantitative interpretation. In summary, while Cu remains the ideal choice for CO_2_ electroreduction, its dependence on high overpotential continues to limit practical deployment. Advancing Cu‐based catalysts that deliver high efficiency at low energy input is therefore essential for making this process viable.

### 4.3 Single Atom Catalysts

In metal nanoparticle catalysts, catalytic activity is governed by surface facets, edges, and metal‐support interfacial sites, which collectively regulate the adsorption and activation of reactants and intermediates [139]. In contrast, when metals are dispersed as isolated single atoms on supports, these nanoparticle‐specific structural features, such as crystal facets, particle size effects, edges, and corner atoms, are eliminated, fundamentally altering the catalytic behavior. These unique catalysts, commonly called Single‐atom catalysts (SACs), retain structural and compositional tunability comparable to nanoparticle catalysts, while also exhibiting distinct characteristics, including atomically isolated active centers, maximal metal dispersion, and well‐defined local coordination environments [140, 141]. Owing to these advantages, single‐atom catalysts and single‐atom‐site electrocatalysis have emerged as major frontiers in contemporary catalytic research [142, 143, 144].

**FIGURE 7:**
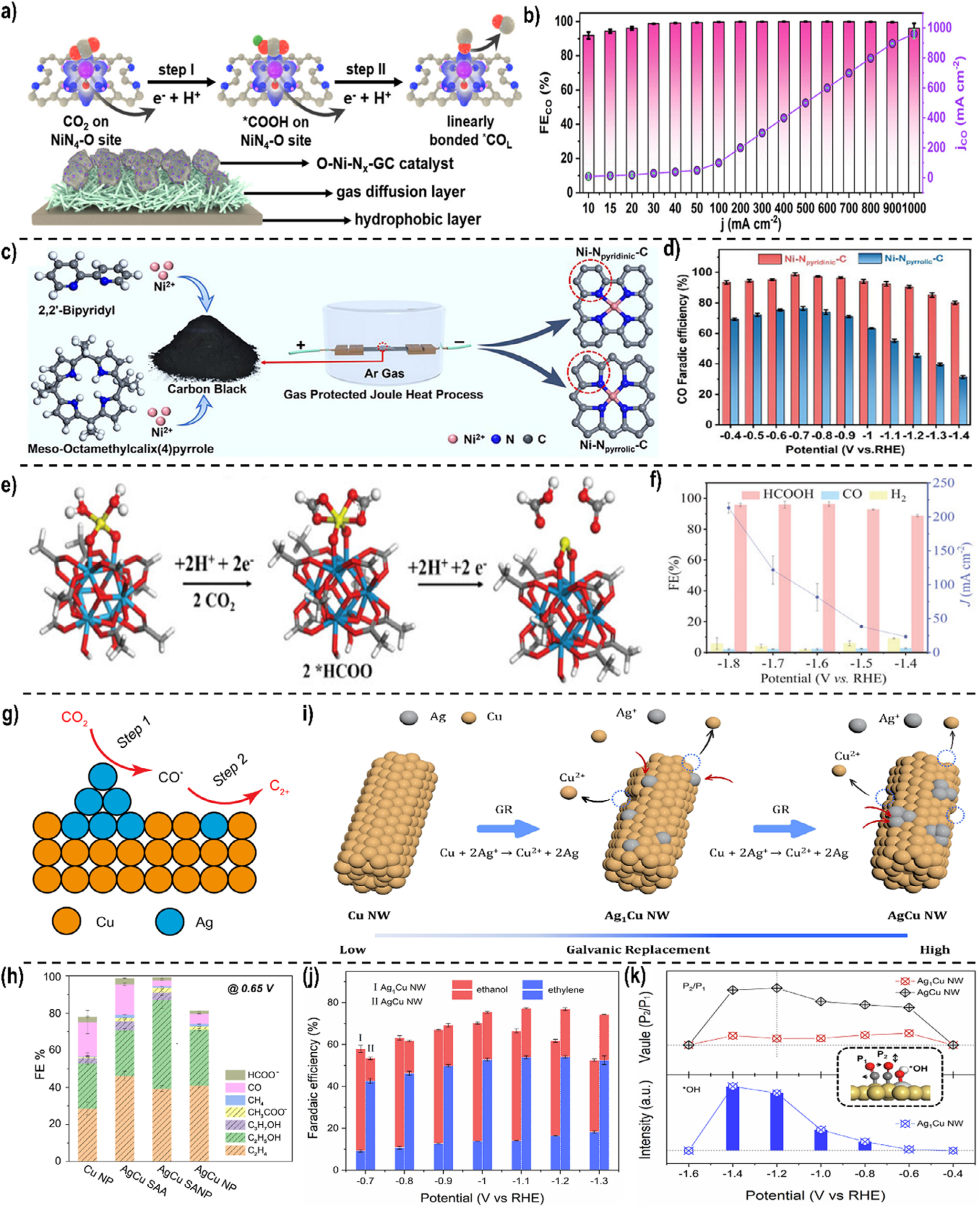
(a) Schematic illustration of the electrocatalytic CO_2_RR mechanism of the O‐Ni‐N_x_ ‐GC system operating in a flow‐cell configuration. (b) F.E. and partial CO current density of O‐Ni‐N_x_ ‐GC measured as a function of applied current density. Reproduced with permission from [145]. Copyright 2025, American Chemical Society. (c) Schematic representation of the synthetic procedures for Ni‐N_pyridinic_ ‐C and Ni‐N_pyrrolic_‐C catalysts. (d) Potential‐dependent F.E. of CO measured on Ni‐N_pyridinic_‐C and Ni‐N_pyrrolic_‐C catalysts. Reproduced with permission from [146]. Copyright 2025, Wiley‐VCH. (e) Proposed electrocatalytic reaction pathway for CO_2_ reduction to HCOOH over the Zr‐BTB‐In catalyst. (f) F.E. of all reaction products obtained using Zr‐BTB‐In as the electrocatalyst. Reproduced with permission from [147]. Copyright 2025, Wiley‐VCH. (g) Schematic illustration of the cascade catalytic mechanism over Ag‐Cu SANP catalysts. (h) F.E. results of Cu NP, Ag‐Cu SAA, Ag‐Cu SANP, and Ag NP catalysts for the CO_2_ reduction reaction at ‐0.65 V. Reproduced with permission from [148]. Copyright 2023, Nature Publishing Group. (i) Schematic representation of the synthesis pathways for Ag_1_Cu and AgCu nanowires, with Ag and Cu atoms depicted in grey and yellow, respectively. (j) CO_2_‐to‐C_2_ products F.E. measured on I) Ag_1_Cu and II) AgCu nanowires in a flow‐cell configuration using 1 M KOH electrolyte. (k) Potential‐dependent Raman analysis showing the intensity ratio of Cu─CO stretching (P2) to Cu─CO frustrated rotation (P1) on AgCu nanowires and single‐Ag‐atom‐modified Cu nanowires, together with the evolution of OH‐related peak intensity as a function of applied potential on Ag_1_Cu NW. Reproduced with permission from [149]. Copyright 2024, Nature Publishing Group.

Among the diverse electrocatalytic CO_2_RR products, CO is a pivotal intermediate, enabling the formation of C_2+_ products, hydrocarbons, and oxygenates, and serving as a primary component of syngas [150, 151]. SACs use almost every atom efficiently and exhibit well‐defined electronic structures in designed coordination environments, making them very promising electrocatalysts for CO_2_RR [152]. However, immobilization of single metal atoms on suitable supports through strong chemical interactions to prevent aggregation driven by high surface free energy, and the stability during electrocatalytic performance critically depend on the nature, density, and coordination environment of anchoring sites within the host matrix [153]. On the contrary, metal‐nitrogen‐carbon (M‐N‐C) catalysts have emerged as leading CO_2_RR materials owing to their low cost, near‐maximal metal utilization, well‐defined active sites, and outstanding chemical and electrochemical stability [154, 155, 156]. Accordingly, Gong et al. introduced a computational intrinsic descriptor (Φ), where Φ = V_M_ × E_M_ / r_M_ (V_M_ denotes the valence electron count, E_M_ the electronegativity of the central metal, and r_M_ the ionic radius). The Φ correlates with the electronic structure of the central metal, thereby providing a predictive parameter for evaluating and rationalizing the catalytic performance of a suitable metal center in the M‐N‐C CO_2_RR system [157]. Given the technoeconomic viability, most M‐N‐C catalysts exhibit limited CO_2_‐to‐CO electrocatalytic performance due to substantially higher overpotentials and compromised stability, ultimately diminishing overall energy conversion efficiency [158, 159]. Conversely, Sun et al. [145] developed atomically dispersed nickel sites with axial oxygen coordination (NiN4‐O) anchored on graphitic N‐rich porous carbon capsules (O‐Ni‐N_x_ ‐GC) as illustrated in Figure 7a. The catalyst delivers nearly quantitative CO selectivity, sustaining ∼100% CO F.E. over a wide range of current density (200–900 mA cm^−2^) and >96% FE_CO_ even at 1 A cm^−2^ (Figure 7b), while exhibiting outstanding durability with stable operation for 140 h at 100 mA cm^−2^ and negligible loss in selectivity (FE_CO_ > 99%). Mechanistic studies combining operando ATR‐SEIRAS spectroscopy with density functional theory (DFT) calculations demonstrate that the outstanding catalytic activity of O‐Ni‐N_x_‐GC arises from synergistic electronic regulation of the Ni center by the axial oxygen ligand and the graphitic nitrogen‐rich carbon support. The axial oxygen ligand enhances electron delocaliszation at the NiN_4_ center, while the graphitic nitrogen matrix downshifts the Ni 3d‐band center, collectively optimizing intermediate adsorption by reducing the Gibbs free‐energy barrier for the rate‐determining *COOH formation to 0.34 eV and weakening *CO binding, resulting in enhancing CO generation and mitigating catalyst poisoning. Both theoretical and experimental studies have established that M‐N_x_C motifs serve as the active centers in single‐atom catalysts, where the spin state governed by the 3d orbital electron configuration, critically dictates intrinsic activity by modulating the electronic structure, tuning intermediate adsorption energies, steering reaction selectivity, [160, 161] instance, Miao et al. [162] validated this by synthesizing a nickel SAC with axial phosphorus coordination (NiP‐N_4_ ‐C), which induces a transition from a low‐spin (S = 0) to a high‐spin (S = 2) state. This electronic reconfiguration, characterized by a structural shift from square‐planar to square‐pyramidal, elevates the energy level of the orbital and significantly enhances the orbital coupling between the Ni center and the reaction intermediates. Consequently, this optimized orbital interaction strengthens the adsorption of the key *COOH species, drastically lowering the Gibbs free energy barrier for the rate‐determining step from 2.06 to 0.70 eV, thereby achieving near‐unity CO selectivity (>99%) and a high turnover frequency of 37.2 s^−1^. However, a clear understanding of how coordinated nitrogen species regulate the spin state and catalytic behavior of single‐atom catalysts remains elusive [163]. Nitrogen functionalities (e.g., pyridinic, pyrrolic, and graphitic N) in M‐N_x_C systems make the realization of uniformly coordinated N environments highly challenging [164, 165]. Regarding this context, Chen et al. [146] utilize a gas‐protected flash Joule heating strategy to synthesize nickel single‐atom catalysts with highly uniform, distinct coordination environments (Ni‐N_pyridinic_‐C and Ni‐N_pyrrolic_‐C) as illustrated in Figure 7c. This precise structural regulation enabled a definitive demonstration that coordinated nitrogen species function as a key switch for the spin state of the metal center, with pyridinic‐N coordination inducing a high‐spin Ni configuration (two unpaired electrons) and delocalized d orbitals, whereas pyrrolic‐N stabilizes a low‐spin state. The pyridinic‐N‐driven high‐spin configuration enhances d‐p orbital coupling between the Ni dz^2^ orbital and the CO_2_ π* orbital, thereby strengthening adsorption of the rate‐determining *COOH intermediate and reducing the associated Gibbs free‐energy barrier to 1.57 eV. The system achieved a CO F.E. of 98.8% (Figure 7d) and industrial‐grade partial current densities exceeding 450 mA cm^−2^.

Among main‐group metals, including (In) [166], tin (Sn) [167], antimony (Sb) [168]), and bismuth (Bi) [169] constitute a promising class as electrocatalyst for CO_2_ reduction. In particular, Indium has attracted growing interest owing to its low toxicity and environmental compatibility. Nevertheless, indium single‐atom catalyst (SACs)‐based systems generally suffer from diminished F.E., limited current density, and poor stability at highly cathodic potentials, substantially restricting their practical deployment under neutral and alkaline conditions [170, 171]. Although acidic CO_2_ reduction systems suppress carbonate formation and enhance CO_2_ utilization efficiency, they are challenged by competitive hydrogen evolution, and, critically, inadequate catalyst stability [172]. Conversely, Li et al. [147] engineered tetrahedrally coordinated In (III) single‐atom sites stabilized within a two‐dimensional Zr‐based metal‐organic framework (Zr‐BTB), for electrochemical CO_2_R in an acidic environment. Notably, within the Zr_6_ clusters, two pairs of Zr ions spaced by 3.30 Å are each coordinated by monodentate hydroxyl groups, which can act as chelating anchors for indium ions to generate isolated single‐atom sites. The Fourier‐transformed EXAFS spectrum corroborates a dominant peak at 1.66 Å, attributable to In‐O scattering, and quantitative fitting yields a coordination number of four, confirming atomically dispersed In species in a tetrahedral four‐oxygen‐atom coordination environment. The Zr‐BTB‐In catalyst exhibits outstanding formic acid electrosynthesis with a F.E. of 95.7% and a current density of 213.3 mA cm^−2^ at −1.8 V versus RHE (at pH 1.67) (Figure 7f), while maintaining exceptional stability in acidic media by sustaining ∼100 mA cm^−2^ for over 20 h without noticeable performance degradation. Moreover, the authors utilized a membrane electrode assembly electrolyzer incorporating a solid‐state electrolyte (MEA‐SSE) to enable the direct generation of high‐purity formic acid. Operating at a cell voltage of 4.0 V, the electrolyzer delivered a current density of ∼102.0 mA cm^−2^ and continuously produced aqueous formic acid with 100% relative purity, achieving an ultrahigh concentration of 505.5 mmol L^−1^, approximately 1.5 times higher than that of state‐of‐the‐art catalysts, [173, 174, 175] with sustaining stable performance for more than 10 h. Combined operando ATR‐FTIR spectroscopy and DFT analyses reveal that the exceptional CO_2_RR activity of Zr‐BTB‐In originates from a distinctive dual‐pathway mechanism (Figure 7e) operating at low‐coordination indium single‐atom sites. Spectroscopic analysis traces the reaction pathway via the identification of characteristic *COOH (1385 cm^−1^) and *CO_2_^−^ (1540 cm^−1^) intermediates, while thermodynamic calculations confirm indium sites as the dominant active centers with a substantially reduced energy barrier (ΔG_max_ = 0.89 eV) relative to Zr nodes. Notably, the large ionic radius of In^3+^ (0.92 Å) enables a unique coordination geometry that accommodates simultaneous binding of two CO_2_ molecules, yielding a stable octahedral complex in which both *HCOO intermediates chelate the metal center. This dual‐molecule activation mechanism markedly lowers the rate‐determining free‐energy barrier to 0.49 eV, resulting in a calculated ∼1.5‐fold enhancement in the reaction kinetics for formic acid production.

Heterometallic single atoms are pivotal in Cu‐based CO_2_R systems by disrupting the intrinsic kinetic scaling relationships of pristine Cu‐systems through the creation of tailored atomic‐scale environments that regulate intermediate adsorption and reaction pathways. Accordingly, Du et al. [176] investigated Cu‐based alloy catalysts incorporating p‐block metals, utilizing host‐guest metal interactions [177, 178, 179] in two inverse single‐atom alloy (SAA) configurations to selectively modulate the reaction pathway. The Cu‐In SAA design strategy was based on substituting the guest single‐atom metal within the host metal matrix. Theoretical data within situ ATR‐SEIRAS validation suggest that an In single‐atom in Cu matrix (Cu_99_In_1_) induces weakened monodentate *CO absorption energy, resulting in high CO F.E. surpassing 90%. Conversely, the Cu single atom in the Indium matrix (Cu_1_In_99_) has stabilized bidentate *CO and HCOO* absorption, favoring selectivity of HCOOH production with F.E. over 90%. From a structural and mechanistic perspective, the incorporation of single heteroatoms or heteroatom nanoparticles induces asymmetric coordination motifs (through lattice strain) within the Cu framework, resulting in enhancing *CO binding on neighboring Cu sites and steering subsequent hydrogenation steps followed by C─C coupling, to tune selectivity between specific C_2_ products (ethanol/ethylene) [135, 180]. In this context, Du et al. [148] developed a cascade Ag‐Cu electrocatalyst, via a galvanic replacement strategy [181], comprising isolated Ag single atom alloy (Ag‐Cu SAA) and Ag single atoms with nanoparticles (NPs) (Ag‐Cu SANP) within a commercially available Cu NPs system. Ag is a well‐known catalyst for the selective CO_2_R to CO, with Ag active sites, either as isolated atoms in Ag‐Cu SAA or as nanoparticles in Ag‐Cu SANP systems [182, 183]. Employing DFT study, it is observed that in Ag‐Cu SANP system (where EXAFS analysis indicates an Ag─Cu to Ag─Ag bond ratio of approximately 1:7), the introduction of Ag single atoms into Cu nanoparticles induces asymmetric Cu‐Ag coordination and local compressive strain (shortening adjacent Cu─Cu bonds from 2.57 to 2.50 Å), enhancing *CO adsorption on Cu sites and thereby significantly steering C─C coupling activity. The schematic mechanistic pathway illustrated in Figure 7g. Figure 7h presents that the resulting Ag‐Cu SANP catalyst delivered a F.E. of 94 ± 4% toward multicarbon products at ‐0.65 V, markedly outperforming the reference catalysts (56% for Cu NPs and 78% for Ag‐Cu SAA). Acetate is also formed only in minor amounts because its formation pathway diverges from the common *CO‐CHO intermediate and incurs a thermodynamic penalty of 0.19 eV relative to the energetically favored *OCH‐CHO intermediate that leads preferentially to ethylene and ethanol. However, this integrated cascade architecture exposes a fundamental trade‐off: although it optimizes overall energy efficiency, it produces a mixed ethylene‐ethanol product distribution because the common electronic modulation lowers the activation barriers of both reaction pathways to nearly identical values (∼ 0.04 eV difference), leading to comparable reaction probabilities rather than selective formation of a single product. Moreover, this earlier study couldn't confirm the absolute role of Ag single atoms and Ag nanoparticles for tuning the selectivity pathway between ethanol and ethylene. Subsequently, Wang et al. [149] introduced Cu nanowires (NWs) decorated with Ag NPs (AgCu NWs) and Cu NWs decorated with Ag Single Atoms (Ag1Cu NWs) by a galvanic replacement approach (Figure 7i) to decipher and control specific reaction pathways. The single‐atom Ag_1_Cu NW catalyst achieves a dominant F.E. of 56.3% for ethanol at −1.0 V vs. RHE, raising the ethanol‐to‐ethylene ratio to 4.26 compared to just 0.41 on the nanoparticle counterpart, while the AgCu NW catalyst favours ethylene production (54.9%) at −1.1 V vs. RHE (Figure 7j). Operando Raman spectroscopy collected under electrocatalytic conditions shows that the AgCu NW catalyst exhibits three distinct vibrational features attributable to adsorbed *CO species: at 282 (frustrated rotation Cu‐CO denoted as P1), 362 (Cu─CO stretching denoted as P2) and 1950–2150 cm^−1^ (C─O stretching) [184]. Figure 7k presents the potential‐dependent P2/P1 intensity ratios for AgCu NW and Ag_1_Cu NW, where the consistently higher ratios observed on AgCu NW indicate elevated *CO surface coverage, favouring *CO‐*CO coupling [99, 185] Corroborated with operando ATR‐SEIRAS measurements, the emergence of an absorption band at 1561 cm^−1^ (*CO‐*CO intermediate), at −0.4 V vs. RHE, with its intensity peaking at ‐1.2 V versus RHE; this trend closely tracks the ethylene F.E., indicating that *CO‐*CO plays a key role in ethylene formation on AgCu NW [186]. In contrast, operando Raman spectra of Ag_1_Cu NW show a distinct band at 537 cm^−1^ assigned to surface *OH species, [187] appearing at −0.6 V vs. RHE and intensifying at more negative potentials, indicating that isolated Ag sites not only facilitate *CO formation but also promote *CO hydrogenation to *CHO through in situ‐generated *OH. Operando ATR‐SEIRAS shows that the intensity of the *COCHO band at 1083 cm^−1^ increases with applied potential from −0.4 to −1.2 V vs. RHE, accompanied by a concomitant decrease in the *CHO band at 1244 cm^−1^, [151] indicating the formation of *COCHO via asymmetric *CO‐*CHO coupling on adjacent Cu sites (HO‐Ag_1_‐Cu‐Cu), which preferentially drives CO_2_ reduction toward ethanol.

### 4.4 Molecular Catalyst

Molecular catalysts are well‐defined metal‐ligand complexes comprising single or multiple metal centers coordinated by organic ligands, offering highly tunable platforms for electrochemical transformations such as carbon dioxide reduction (CO_2_RR). In electrocatalysis, their well‐defined architectures enable precise tuning of the metal center's electronic properties and second coordination sphere through rational ligand design, thereby affording high activity and selectivity toward targeted products spanning C_1_ species to multicarbon (C_2_‐C_3_) hydrocarbons and alcohols [21, 191].

**FIGURE 8:**
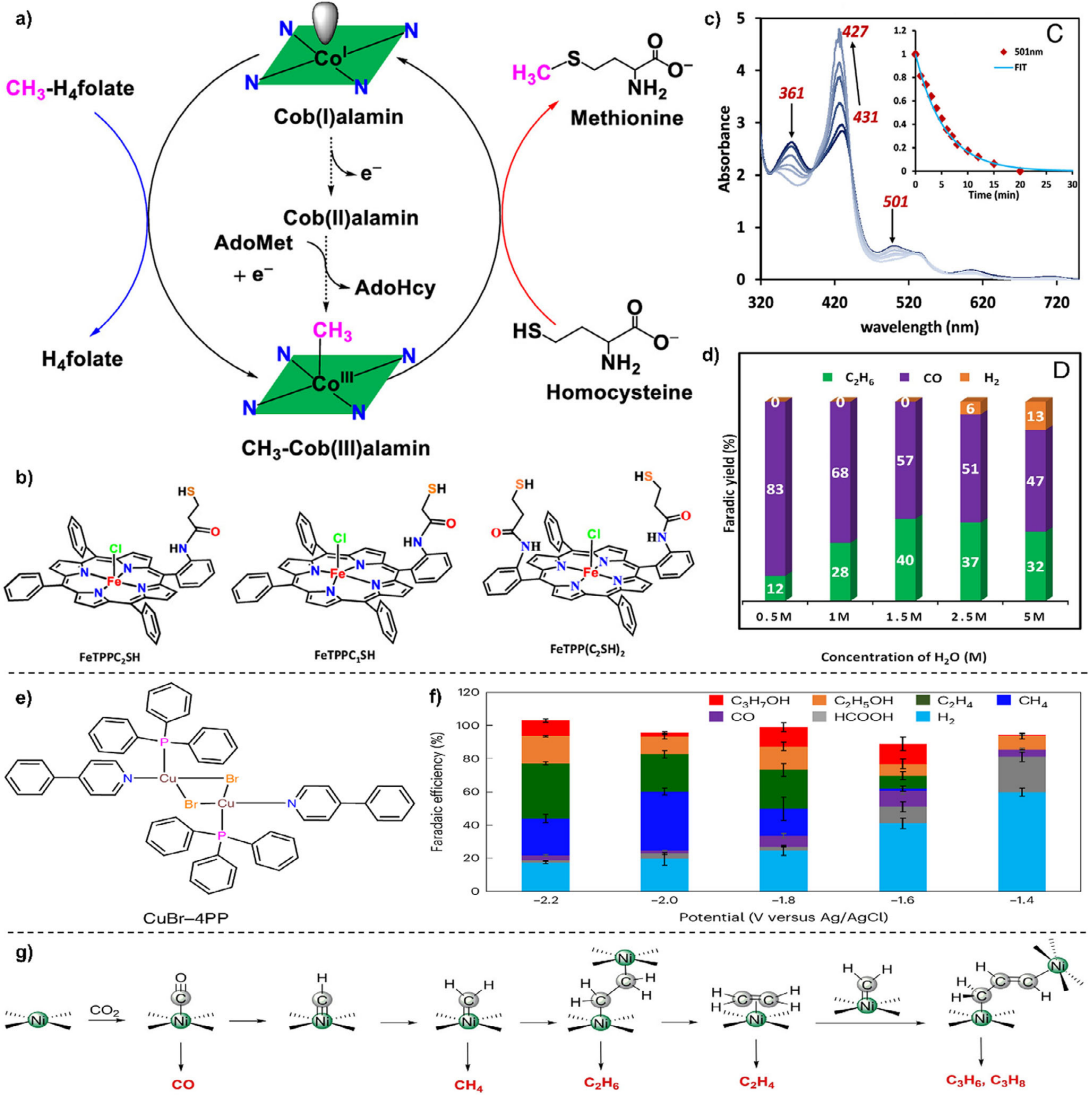
(a) Schematic representation of the methyl‐transfer mechanism in methionine synthase. (b) Chemical structures of FeTPPC_2_SH, FeTPPC_1_SH, and FeTPP(C_2_SH)_2_. (c) UV‐vis evidence for the time‐dependent decay of the Fe(II)‐CH_2_ species to free Fe(II) facilitated by a second‐sphere thioether moiety (inset: kinetic analysis). (d) Faradaic product distributions at varying proton concentrations. Reproduced with permission from [188]. Copyright 2025, National Academy of Sciences. (e) Structure of CuBr‐4PP copper‐bromide phosphine complexes. (f) Product F.E. after 1 h of CO_2_ electroreduction on CuBr‐4PP in 0.5 M KHCO_3_. Reproduced with permission from [189]. Copyright 2024, Nature Publishing Group. (g) Proposed CO_2_ electroreduction pathways for square‐planar nickel thiolate complexes. Reproduced with permission from [190]. Copyright 2023, Wiley‐VCH.

In monometallic CO_2_ reduction catalysts, metal‐bound methylene and methyl intermediates are likely formed, but in the absence of a suitable “parking site” within the catalyst architecture, these species typically terminate as C_1_ products such as CH_3_OH or CH_4_ [192, 193, 194, 195]. A biological solution to this limitation is illustrated by enzyme methionine synthase (Figure 8a), where a thiol group from homocysteine transiently accepts a ─CH_3_ group from a Co(III)‐corrin cofactor. By analogy, introducing thiol functionalities near an iron porphyrin active site could provide a temporary methyl acceptor, enable C─C coupling and thus facilitate the formation of C_2_ products during CO_2_ reduction. Capitalizing on this, Dey et al. have introduced pendant thiols in the second sphere of the iron porphyrin molecular catalyst (Figure 8b) [188]. These pendant thiols act as “parking space”, where iron porphyrin carbene (Fe═CH_2_) (fist carbon molecule of CO_2_) is parked by the attack of the nucleophilic thiol to transfer the carbon fragment and form a pendant methyl thioether, similar to a study as reported earlier by Tyagi et al. [196] By parking of the initial C_1_ intermediate, the pendent thiol group suppresses its early dissociation as methane or methanol. This stabilization allows the iron center in its more oxidative (Fe^0^) state to activate a subsequent CO_2_ molecule and, through a sequence of proton‐coupled electron‐transfer steps, generate an Fe(II)‐CH_3_ intermediate. The C─C bond formation proceeds via heterolytic cleavage of the Fe─C bond in the Fe(II)‐CH_3_ intermediate, which couples with the “parked” methyl group to form C_2_H_6_. This step restores the catalyst to its active state, allowing subsequent catalytic cycles [188]. This coupling mechanism and methyl‐transfer pathway are further supported by studies with a modified catalyst in which a pendant ethyl thioether (‐SC_2_H_5_) is introduced into the second coordination sphere. Chemical generation of an Fe(II)‐CH_3_ species in this framework leads to direct coupling between the iron‐bound methyl group and the “parked” ethyl group, producing propane (C_3_H_8_). This process is corroborated by UV‐vis spectroscopy, which shows decay of the Fe(II)‐CH_3_ intermediate to a free Fe(II) species (Figure 8c). The architecture of the catalyst's secondary coordination sphere is crucial for C‐C bond formation; a single thiol functionality is more effective than two. Accordingly, FeTPPC_2_SH outperforms FeTPP(C_2_SH)_2_, which shows a reduced ethane Faradaic yield (FY) from 40% to 34% under identical conditions, indicating that a single biomimetic thiol provides the most favourable spatial arrangement for C─C coupling. Proton availability, supplied by H_2_O, also strongly influences product selectivity. At low proton concentrations, the reaction favours 2e^−^/2H^+^ reduction to CO, whereas increasing the proton concentration to an optimal 1.5 M redirects the pathway toward ethane formation, achieving a maximum FY of 40% (Figure 8d). At higher proton concentrations (e.g., 2.5–5 M), the hydrogen evolution reaction becomes dominant, generating H_2_ and suppressing ethane selectivity.

Most molecular CO_2_ reduction catalysts reported to date are mononuclear, for which the low intrinsic reactivity of CO_2_ limits activation to a single metal center and makes C─C coupling unlikely [197, 198]. Consequently, beyond C_2_ formation is generally proposed to require multinuclear catalyst architectures [199, 200] capable of strongly retaining CO_2_ and key intermediates beyond physisorption, through ligand functionalities such as amines or π‐conjugated groups to lower activation barriers and enhance selectivity [201, 202]. Although such systems often suffer from rapid degradation (metal cluster formation) under dynamic conditions [203, 204]. Accordingly, Sakamoto et al. designed Cu‐Br bridged complexes, among them di‐nuclear Cu_2_(µ‐Br)_2_ core coordinated by triphenylphosphine and 4‐phenylpyridine ligands (CuBr‐4PP), which outperformed di‐nuclear CuBr‐12B and trinuclear CuBr‐BisM (Figure 8e). Both the trinuclear CuBr‐BisM and the di‐nuclear control CuBr‐12B complex exhibited poor stability and low selectivity toward multicarbon products, attributable to their limited ability to retain CO_2_ and to LUMO distributions partially localized on the Cu‐Br core. Employing XAFS and DFT, this electronic structure facilitated rapid decomposition into catalytically inactive Cu (0) aggregates, underscoring that increased nuclearity alone is insufficient to enable C─C coupling without precise electronic tuning and stabilization of active sites via metal‐ligand charge‐transfer interactions under catalytic conditions. In contrast, the CuBr‐4PP catalyst incorporated 4‐phenylpyridine ligands that shifted LUMO localization onto the ligand framework and induced pronounced CO_2_ adsorption–desorption hysteresis (retaining a high local concentration of substrate around the di‐nuclear active sites). Operando XAFS measurements demonstrated that CuBr‐4PP preserves its Cu(I) oxidation state and molecular integrity, whereas the CuBr‐12B control, which undergoes reduction to metallic Cu (0) aggregates. Corroborated with DFT calculations, attribute this stability to the 4‐phenylpyridine ligands, which function as electronic buffers by delocalizing excess charge into their π‐systems while permitting adaptive Cu─Cu distance to accommodate bridging reaction intermediates. Consequently, a potential of −2.2 V (vs. Ag/AgCl), CuBr‐4PP attained a total F.E. for C_2+_ products of approximately 60%, with specific efficiency for n‐propanol reaching ∼10%–12% (Figure 8f). The C_3_ product formation mechanism involves a dynamic di‐nuclear C─C coupling pathway, initiated by CO insertion into the Cu‐Br‐Cu bridge that expels the labile bromide ligand and generates a [Cu‐CO‐Cu] ^+^ intermediate (ν^12^_CO_ =  2,126 cm^−1^). Successive proton‐coupled electron transfer steps transform this species into a bridging aldehyde‐like intermediate ([Cu‐CHO‐Cu] ^+^), which enables insertion of a second CO to generate the C_2_ intermediate [Cu‐CHOCO‐Cu]. This inherent flexibility of the di‐nuclear framework allows the bridging intermediate to migrate to one Cu center while the other binds an additional substrate, thereby permitting sequential insertion of a third CO to form the C_3_ precursor [Cu‐CHOCOHCO‐Cu], as observed in the 1 540 to 1 560 cm^−1^ range in an in situ SERS study [189].

To address non‐Cu system efficiency and the persistent instability of conventional Cu‐based catalysts, for C_2+_ product formation, Du et al. adopted a bio‐inspired [Ni‐Fe] hydrogenases and carbon monoxide dehydrogenases (CODH) pathway [205], utilizing molecular nickel thiolate complexes (square‐planar to octahedral) to achieve CO_2_ reduction to C_3_ hydrocarbons. Among the five molecular complexes evaluated, complexes 1 and 2 show no catalytic activity, as evidenced by linear sweep voltammetry, which reveals no appreciable current enhancement under CO_2_‐saturated conditions. While Complex 3 (Ni(mpo)_2_, mpo = 2‐mercaptopyridyl‐N‐oxide) can produce C_1‐3_ hydrocarbons, its unique ligand environment causes a distinct selectivity shift when CO is added to the system, resulting in the FE for HCOOH rises significantly from 3.6% to 12.1% in the presence of CO. The specific electronic or steric properties of the 2‐mercaptopyridyl‐N‐oxide ligand in Complex 3 appear to channel this Ni‐CO intermediate toward the formate pathway, rather than facilitating the deep reduction and intermolecular coupling required for C─C bond formation. Complex 4 (Ni(pyS)_3_; pyS = 2‐mercaptopyridine), an octahedral species, undergoes electrochemically induced dechelation, evidenced by two reduction waves in cyclic voltammetry prior to catalytic onset, thereby generating a coordinatively unsaturated Ni center capable of CO_2_ binding. Although this activation enables C_1_‐C_3_ hydrocarbon formation, the associated energetic and kinetic penalties lead to reduced catalytic efficiency and lower baseline F.E. relative to pre‐organized square‐planar analogue (e.g., Complex 5). When CO feed is added directly to the system (bypassing the initial CO_2_ activation and facilitating the Ni‐CO intermediate), the yield of all hydrocarbon products from Complex 4 is significantly boosted. This implies that once the structural barrier of dechelation is overcome and the intermediate is formed, the resulting species is highly active. Complex 5 ((Ni(mp)_2_, mp = 2‐mercaptophenolate)) achieved an F.E. of 8.2% specifically for C_3_ products, and the total FE for all C1‐3 hydrocarbons reached 26.0% in pH‐neutral aqueous solution. In the presence of CO feed, the formation of a critical Ni‐CO intermediate is evidenced by a significant increase in F.E. (up to 41.1%). Following the initial reduction of CO_2_ to the adsorbed Ni‐CO species, the cycle proceeds through sequential proton‐coupled electron transfers to generate nickel‐methylidyne (Ni≡CH) and nickel‐methylidene (Ni = CH2) intermediates. Because the catalyst functions as a discrete monometallic species physically adsorbed on the electrode, C─C bond formation is proposed to occur via intermolecular coupling between adjacent nickel centres. Specifically, the coupling of two Ni‐methylidene species yields C_2_ products (ethane and ethylene), while the unprecedented formation of C_3_ hydrocarbons (propane and propylene) is attributed to a secondary coupling step between a nickel‐olefin intermediate and a nickel‐methylidyne species (Figure 8g), a pathway mechanistically analogous to ruthenium‐catalysed olefin metathesis [206]. Moreover, the molecular integrity of Complex 5 (Ni(mp)_2_) was unequivocally established by post‐electrolysis analyses. Employing X‐ray photoelectron spectroscopy showed unchanged Ni 2p binding energies (854.7 and 872.2 eV) before and after electrolysis, excluding the formation of NiO or NiS, while HR‐TEM and DLS measurements verified the absence of nickel nanoparticle formation on the electrode or in the electrolyte [190].

### 4.5 Emerging Electrocatalysts: High‐Entropy Materials

**FIGURE 9:**
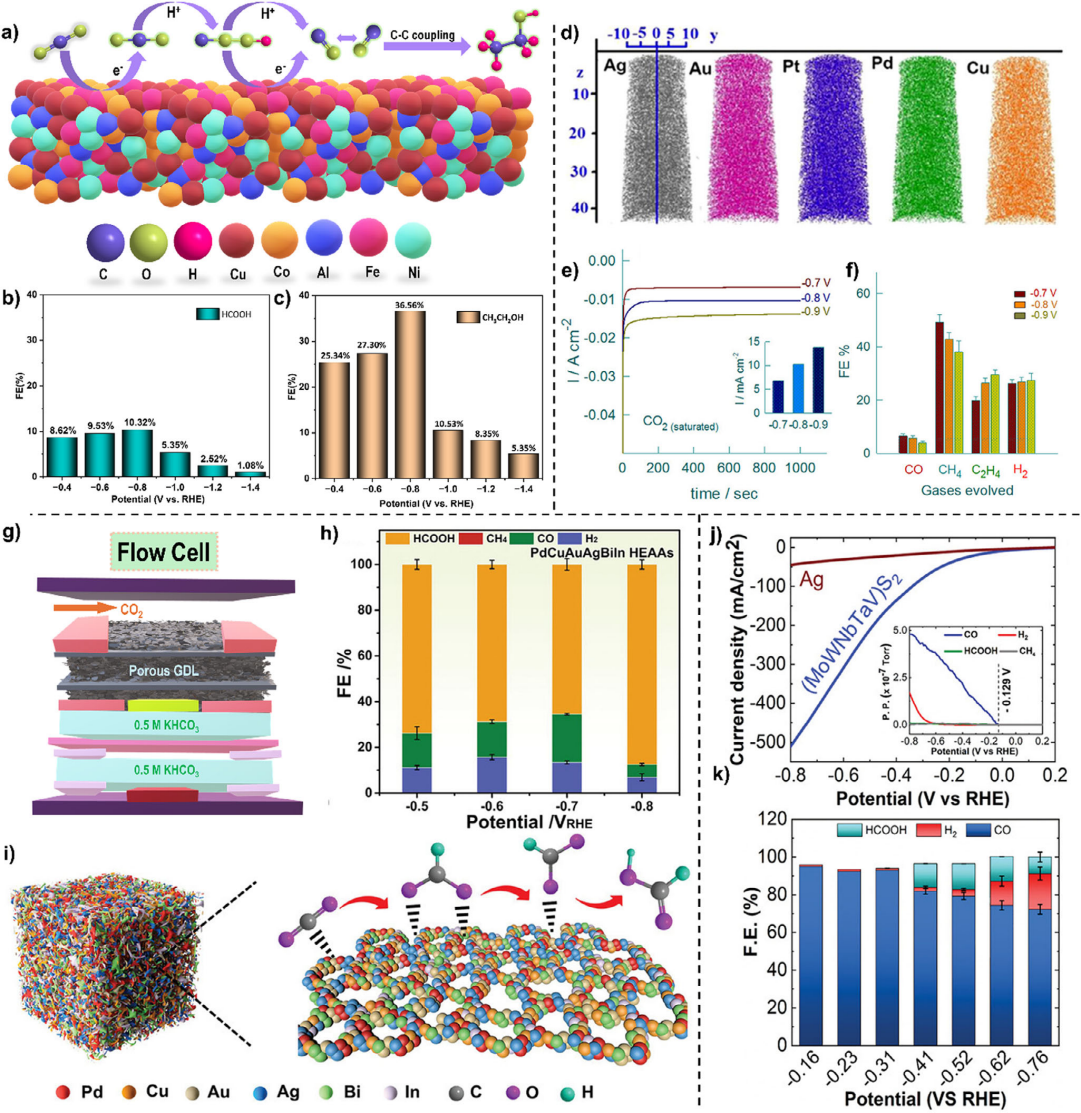
(a) Schematic illustration of CO_2_ reduction pathways on the CuCoAlFeNi HEA catalyst. Liquid‐product FE for (b) Cu and (c) CuCoAlFeNi high‐entropy alloy measured in an H‐type cell with a CO_2_ flow rate of 10 sccm. Reproduced with permission [207]. Copyright 2025, Royal Society of Chemistry. (d) Elemental homogeneity of Au, Ag, Pt, Pd, and Cu as revealed by atom probe tomography mapping. (e) Chronoamperometric responses in CO_2_‐saturated electrolyte at −0.7, −0.8, and −0.9 V over 1000 s, with corresponding current densities shown in the inset. (f) FE (with error bars) for carbon‐containing products and H_2_ at each potential. Reproduced with permission [208]. Copyright 2020, American Chemical Society. (g) Schematic diagram of the flow‐cell setup. (h) FE CO_2_ reduction products in the flow cell over PdCuAuAgBiIn HEAAs as a function of applied potential. (i) Schematic depiction of enhanced HCOOH production over PdCuAuAgBiIn HEA aerogels. Reproduced with permission [209]. Copyright 2023, Wiley‐VCH. (j) Current density of (Mo, W, V, Nb, Ta) and Ag nanoparticles as a function of potential versus RHE obtained from LSV, with the corresponding gaseous product partial pressures shown in the inset. (k) FE of CO, H_2_, and HCCOH from chronoamperometry measurements on (Mo, W, V, Nb, Ta) nanoflowers at various applied potentials. Reproduced with permission [210]. Copyright 2021, Wiley‐VCH.

High‐entropy alloys (HEAs), composed of five or more metallic elements in near‐equiatomic ratios, are emerging as a transformative class of catalytic materials. Their inherently complex atomic structures give rise to diverse local environments, tunable electronic states, and abundant active sites, collectively endowing them with remarkable catalytic potential. Despite this promise, the catalytic applications of HEAs remain relatively unexplored, offering vast opportunities to redefine catalyst discovery by intelligently navigating their immense compositional and structural space [211, 212]. Xing et al. synthesised a CuCoAlFeNi HEA via a solvothermal approach and systematically evaluated its electrocatalytic performance toward CO_2_RR in a standard three‐electrode setup. As illustrated in Figure 9a, the CuCoAlFeNi HEA follows a multi‐step CO_2_RR pathway that selectively yields ethanol as the major product. Under an applied potential of −1.2 V vs. RHE in CO_2_‐saturated 1 M KHCO_3_, the HEA catalyst achieved a current density of −102.4 mA cm^−^^2^, substantially higher than that of Cu nanoparticles (−66.2 mA cm^−2^), demonstrating superior catalytic activity [207].

Electrolysis experiments conducted in an H‐type cell (CO_2_ flow rate: 10 sccm; applied potentials: −0.4 to −1.4 V vs. RHE) revealed pronounced hydrogen evolution on the Cu nanoparticle electrode, which was effectively suppressed on the HEA catalyst. For liquid‐phase products, the Cu nanoparticle system exhibited a modest FE of 10.32% for HCOOH at −0.8 V vs. RHE (Figure 9b). In contrast, the CuCoAlFeNi HEA exclusively produced ethanol with a significantly enhanced FE of 36.56% (Figure 9c). These results highlight the CuCoAlFeNi HEA as a highly selective and efficient electrocatalyst for CO_2_RR, capable of steering product pathways toward multi‐carbon alcohols while mitigating competing hydrogen evolution.

Biswas's group developed a single‐atom catalyst based on a high‐entropy alloy composed of 5 active metals (Ag, Au, Cu, Pt, and Pd), in which isolated Cu atoms are stabilised by the surrounding metals on an FCC facet. The strong synergistic interactions between Cu and the neighbouring elements are thought to be critical in enhancing the structural and electrochemical stability of the active sites. Atom probe tomography (Figure 9d) confirmed a homogeneous atomic‐level distribution of all constituent elements within a single‐phase FCC matrix, indicating true atomic mixing and alloy formation. Unlike most electrochemical CO_2_RRs, which are typically carried out in a basic electrolyte, the HEA catalyst showed no activity in CO_2_‐saturated 0.5 M NaHCO_3_. The reason for this behavior is presently unknown, though it may be attributed to HEA's pH‐dependent activity. Figure 9e shows the steady‐state current responses of HEA in CO_2_‐saturated electrolyte at −0.7, −0.8, and −0.9 V, yielding current densities of −6.83, −10.31, and −13.81 mA cm^−^^2^, respectively. The inset highlights the notably high CO_2_ reduction activity at −0.9 V. The main gaseous products were CO, CH_4_, C_2_H_4_, and H_2_‐consistent with Cu‐based catalysts. Despite the multielement composition of HEA, only Cu exhibited direct catalytic activity, while other elements contributed synergistically. As shown in Figure 9f, the FE for CO decreased from ∼7% at −0.7 V to 4% at −0.9 V, whereas CH_4_ and C_2_H_4_ formation dominated, reaching FEs of 49.4% and 19.9% at −0.7 V and 38.0% and 29.5% at −0.9 V. The FE for H_2_ remained steady at ∼26%. Notably, the total FE reached ∼100% for gaseous products at −0.9 V (vs. Ag/AgCl), indicating negligible liquid product formation and underscoring the distinct catalytic behavior of HEA [208].

In order to harness the combined advantages of HEAs and aerogels for catalytic applications, Li et al. engineered a PdCuAuAgBiIn HEA aerogel (HEAAs) via a freeze‐thaw synthesis approach. The surface functionality and electrocatalytic performance of the as‐synthesised HEAA were compared with the PdCuAuAgBiIn particles (HEAPs) and Pd metallic aerogels. The HEAAs exhibit a higher density of unsaturated Pd sites than the HEAPs, providing more active centres for the activation of small molecules and, consequently, enhancing catalytic performance. The HEAAs exhibit outstanding CO_2_RR performance, enabling highly selective production of HCOOH and other C_1_ products over a wide potential window. Nearly 100% F.E. for C_1_ products is achieved between −0.7 and −1.1 V_RHE_, with a maximum HCOOH FE of 98.1% at −1.1 V. The electrocatalytic CO_2_RR performance of the PdCuAuAgBiIn HEAAs was further evaluated in a flow‐cell configuration to assess their feasibility for practical industrial applications (Figure 9g). The polarisation curve of the PdCuAuAgBiIn HEAAs shows a high current density of approximately 200 mA cm^−^^2^ at −2.0 V_RHE_. At these potentials, the FE_HCOOH_ of the PdCuAuAgBiIn HEAAs exceeds 50%, reaching a maximum of 87.5% at −0.8 VRHE (Figure 9h). This performance significantly surpasses that of HEAPs and Pd metal aerogels. Furthermore, when implemented in a flow‐cell configuration using 0.5 M KHCO_3_, the catalyst delivers a high current density of ∼200 mA cm^−^^2^ while maintaining an HCOOH FE of 87%. The exceptional activity and selectivity are due to strong synergistic interactions among the constituent metals and the presence of surface‐unsaturated sites in the PdCuAuAgBiIn HEAAs. These characteristics tune the electronic structures of the active metals and optimize the adsorption of the key HCOO* intermediate, thereby enhancing HCOOH formation while effectively suppressing CO poisoning (Scheme, Figure 9i) and competing HER [209].

To understand the functionality of 2D high‐entropy transition metal dichalcogenides (TMDCs), Cavin et al. predicted, synthesised, and characterised four or five transition metals containing TMDCs across multiple length scales. They evaluated CO_2_ electroreduction using (Mo, W, V, Nb, Ta) nanoflakes (NFs) as the cathode catalyst and benchmarked the performance against Ag NPs under identical conditions and comparable particle sizes. The tests were carried out in a CO_2_‐saturated aqueous electrolyte composed of 1 M KOH and 1 M choline chloride, chosen for its high ionic conductivity, suppression of HER, and stability over the applied potential window. All measurements were conducted in a custom two‐compartment cell separated by a Nafion membrane, using a three‐electrode configuration with Ag/AgNO_3_ as the reference and Pt as the counter electrode. Linear sweep voltammetry (Figure 9j) at 50 mV s^−^^1^ on NFs and Ag NPs supported on a gas diffusion layer revealed that, at −0.8 V versus RHE, the (Mo, W, V, Nb, Ta) nanoflakes exhibit a current density much higher than Ag NPs, reaching ≈510 mA cm^−^^2^ (geometric area‐normalised). This five‐metal alloy (MoWVNbTa)S_2_, which exhibits the highest configurational entropy, delivers outstanding CO_2_‐to‐CO electroreduction performance, achieving a 0.51 A cm^−^^2^ current density and 58.3 s^−^^1^ turnover frequency at approximately −0.8 V. First‐principles calculations attribute the enhanced activity to multi‐site catalysis enabled by atomic‐scale disorder, which optimizes the rate‐limiting CO desorption step by generating isolated transition metal edge sites with relatively weak CO binding. Figure 9k indicates that the FE for CO production reaches about 95% at −0.16 V versus RHE and remains above 92% at −0.23 and −0.31 V. Beyond approximately −0.4 V, the CO selectivity declines, dropping to 72% at −0.76 V. In contrast, hydrogen evolution is minimal at potentials up to −0.4 V but increases at more negative potentials, reaching roughly 18% at −0.76 V. Liquid products were analysed using high‐performance liquid chromatography. HCOOH emerged as the primary liquid product beginning at ‐0.4 V. Its average FE was about 13% between −0.4 and −0.61 V, decreasing slightly to around 9% at −0.76 V. These results establish 2D high‐entropy TMDC alloys as a versatile platform for designing high‐performance electrocatalysts for CO_2_ reduction and related electrochemical reactions [210].

Despite numerous reports suggesting a direct correlation between mixing entropy and the exceptional electrocatalytic performance of HEAs, the intrinsic activity of any alloy is fundamentally governed by two competing factors: beneficial ligand interactions, which enhance activity, and the statistical dilution of active sites, which suppresses it [213]. At modest levels of compositional complexity, introducing additional elements can enhance activity by perturbing the local coordination environment and thereby optimising adsorbate binding and reaction energetics. However, as the surface becomes increasingly multicomponent, the probability of forming an ideal active ensemble decreases, and the fraction of truly active configurations shrinks, even if a small number of highly active sites persist. Rather than continuing to search for a single “globally optimal” HEA catalyst, efforts in this field should instead focus on elucidating and exploiting the characteristics that are genuinely distinctive to high‐entropy systems. This more holistic perspective emphasises understanding how features such as extreme compositional complexity, local chemical disorder, and multifunctional active sites give rise to behaviors that are inaccessible in conventional alloys, rather than treating HEAs merely as an expansive parameter space for activity optimisation. This, in turn, will pave the way for unambiguous identification of the active sites that truly govern selective CO_2_ electroreduction.

### 4.6 Electrocatalysts beyond Copper

Despite being underexplored for CO_2_RR, non‐Cu electrocatalysts have demonstrated a unique capability to generate higher‐order C_4_‐C_6_ hydrocarbons. The earliest results of a Cu‐free metal catalyst for CO_2_ electroreduction were reported in 2016, when Lewis and co‐workers revealed that a Ni_x_Ga_y_ intermetallic system could promote C─C coupling to form C_2_ products from aqueous CO_2_ with moderate efficiencies [217]. Building on this early work, the field has advanced swiftly toward the design of robust, versatile, and technologically viable non‐Cu catalysts. Alongside Ni, metals such as Ag, Fe, Sn, Co, and Mo have been identified as active CO_2_RR catalysts, where their inherent physicochemical properties dictate key performance‐determining parameters [218, 219, 220].

**FIGURE 10:**
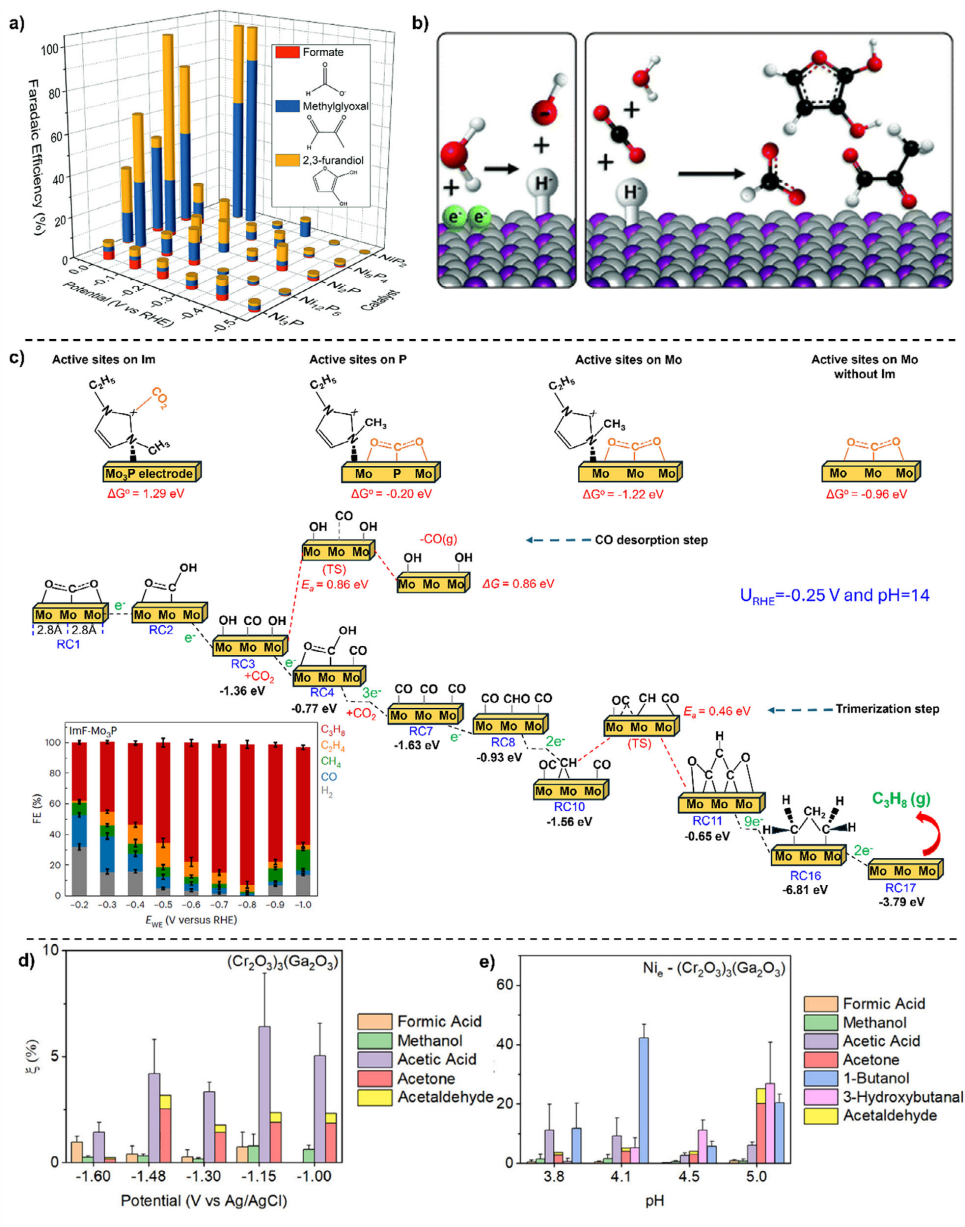
(a) FE for CO_2_RR over NiP_2_, Ni_5_P_4_, and Ni_2_P for the production of 2,3‐furandiol and methylglyoxal. (b) Hydride transfer mechanism in nickel phosphides. Reproduced with permission [214]. Copyright 2018, Royal Society of Chemistry. (c) Proposed reaction pathway for propane formation over ImF‐Mo_3_P (inset: FE of the ImF‐Mo_3_P catalyst evaluated across a range of applied potentials). (d) FE measurements of the ImF‐Mo_3_P catalyst at varying applied potentials. Reproduced with permission [215]. Copyright 2022, Nature Publishing Group. (e) FE of liquid products for (Cr_2_O_3_)_3_(Ga_2_O_3_) in the absence of nickel. Reproduced with permission [216]. Copyright 2023, American Chemical Society.

Transition metal phosphides have attracted significant interest owing to their low overpotential requirements and high selectivity toward energy‐dense products. Calvinho et al. employed five nickel phosphide compounds as electrocatalysts for CO_2_RR, achieving exceptionally high selectivity toward C_1_, C_3_, and C_4_ products. They targeted both *H and *CO binding to promote hydride transfer. Figure 10a shows the FE of the individual products as functions of both potential and catalyst composition. Over 0.05–0.10 V vs. RHE, CO_2_ reduction to 2,3‑furandiol and methylglyoxal dominates on the more phosphorus‐rich nickel phosphides (Ni_12_P_5_, Ni_2_P, Ni_5_P_4_, and NiP_2_), with Ni_2_P affording the highest faradaic yield at the lowest overpotential. In contrast, the low‑phosphorus Ni_3_P exhibits substantially lower CO_2_RR activity relative to HER and inferior selectivity, producing more formic acid than the other catalysts. NiP_2_ achieved a peak methylglyoxal selectivity of 84% at 0.10 V vs. RHE, though testing was limited to potentials no higher than 0.05 V due to the catalyst reaching open‐circuit potential near 0 V, which suppressed current and product formation below detection limits. The highest FE for 2,3‐furandiol (71%) was observed at 0 V vs. RHE on Ni_2_P. Formic acid formation persisted across all potentials but remained below 5% for all catalysts; at more negative potentials (←0.2 V vs. RHE), selectivity shifted toward HER‐unlike on Cu catalysts, where high overpotentials suppress H_2_ evolution and favour CO_2_RR. Moreover, Ni_2_P delivered 4.3 times greater energy efficiency than polycrystalline Cu, likely owing to a hydride‐transfer mechanism (Figure 10b) that enables operation at low overpotential [214].

Asadi et al. adopted a microenvironment modulation strategy by coating MoP with an imidazolium‐functionalized ionomer (Im). They achieved an FE of 77.4% for ethanol at only −0.2 V vs. RHE with a current density of ∼‐90 mA cm^−2^ and excellent long‐term stability, without catalyst morphology restructuring. They further engineered imidazolium‐functionalized Mo_3_P (ImF‐Mo_3_P), where three distinct Mo sites drive CO_2_RR. This outcome was remarkable: propane production reached −395 mA cm^−2^ with 91% FE (inset, Figure 10c), and the performance was sustained for 100 h at −0.8 V vs. RHE, far surpassing that of copper catalysts. Interestingly, pristine Mo_3_P produced only CO and methane, demonstrating that microenvironment engineering, not structure alone, drives multicarbon selectivity. The propane partial current density dropped sharply from ∼100 mA cm^−2^ (ImF‐Mo_3_P) to −1 mA cm^−2^ (Mo_3_P), underscoring the pivotal role of imidazolium functionalization in achieving high current densities. Spectroscopy and DFT analyses reveal that imidazolium ligands reprogram Mo electronic states, promote the formation of stable, multi‐site aggregates of *CO_2_/*CO intermediates, and increase CO trimerisation (Figure 10c). By contrast, Cu modified with imidazolium‐based ionic liquids (OMIm‐Cu_2_O) shows no selectivity improvement, highlighting that even extensive microenvironment engineering cannot overcome the inherent selectivity limitations of Cu [215].

When the desired products extend beyond hydrocarbons to long‐chain oxygenated species, their formation typically follows mechanistically distinct pathways rather than simple CH_x_ insertion processes. In this context, Bocarsly and co‐workers reported the synthesis of 1‐butanol with a FE of up to 42% at −1.48 V vs. Ag/AgCl using a Ni‐based molecular catalyst, Ni_e_(Cr_2_O_3_)_3_(Ga_2_O_3_). Strikingly, the (Cr_2_O_3_)_3_(Ga_2_O_3_) component alone yielded oxalate as the dominant product (≈66%) and showed no formation of C_4_ species. In contrast, incorporation of Ni enabled the selective production of 1‐butanol, clearly identifying Ni as the critical site for effective C─C bond formation (Figure 10d,e). Although trace C_1_ products were detected, the reaction landscape was dominated by C_2+_ species, indicative of efficient and sustained C─C coupling. Mechanistic investigations suggest that (Cr_2_O_3_)_3_(Ga_2_O_3_) facilitates acetaldehyde generation, which was subsequently transferred to Ni sites where aldol condensation and hydrogenation occur. Control experiments further revealed that a nickel coil, despite its substantially higher surface area, produced negligible 1‐butanol compared to a Ni‐electroplated glassy carbon electrode, emphasising the decisive roles of surface structure, crystal facets, and grain boundaries in governing selectivity [216].

Thus, non‐Cu catalysts, though less explored than Cu, can deliver higher‐order C_4_‐C_6_ products, enabled by design principles that go beyond conventional CH_x_‐insertion pathways. Early work on Cu‐free systems, such as NixGay intermetallics, showed that they promote C─C coupling to C_2_ products, which spurred rapid development of more robust and versatile catalysts based on Ni, Ag, Fe, Sn, Co, and Mo. Within this landscape, transition‐metal phosphides (for instance, Ni_2_P and NiP_2_) stand out for combining low overpotentials with high selectivity toward complex oxygenates, where tuning H and CO binding and invoking hydride‐transfer pathways. This allows these materials to outperform polycrystalline Cu in both selectivity and energy efficiency. Building on this, microenvironment engineering, such as imidazolium‐functionalised Mo phosphides, can reprogramme the local electronic structure and stabilise multi‐site *CO_2_/*CO aggregates, unlocking exceptionally high currents and faradaic efficiencies for multicarbon products like ethanol and propane that pristine Mo phosphides or similarly modified Cu cannot match. Finally, Ni‐based molecular systems supported on oxides demonstrate that long‐chain oxygenates, such as 1‐butanol, can be produced through cascade reaction mechanisms. In these systems, the oxide support generates key intermediates (e.g., acetaldehyde), which subsequently undergo aldol condensation and hydrogenation at Ni active sites. Importantly, surface structure, crystal facets, and grain boundaries play decisive roles in governing product selectivity, often exerting a greater influence than metal loading alone. Table 1 summarises the various types of electrocatalysts for CO_2_R, highlighting their selectivity toward specific products along with corresponding Faradaic efficiency, current density, applied potential, and stability.

**TABLE 1:** Recent studies on various type of electrocatalyst for CO_2_RR product selectivity.

Type of catalyst	Catalyst	Product	Faradaic efficiency	Current density & potential	Stability	References
Cu systems	GB‐Cu	Ethylene	38%	37 mA/cm^2^ & −1.2 V vs. RHE	3 h	[221]
	GB‐Cu_29.6_	C_2_^+^ products	73.2%	303.61 mA/cm^2^ & −3.8 V vs. RHE	24 h	[222]
	C‐CuO nanosheets	Ethylene	56.2%	171 mA/cm^2^ & −0.871 V vs. RHE	133 h	[223]
	f‐Cu_2_O	Ethanol	52.6%	9.1 mA/cm^2^ & −0.9 V vs. RHE	45 h	[224]
	c‐Cu_2_O	Ethylene	61.3%	2.5 mA/cm^2^ & −1.2 V vs. RHE	10 h	[225]
	Cu_2_‐CuN_3_	Ethanol	51%	14.4 mA/cm^2^ & −1.1 V vs. RHE	10 h	[226]
	Cu‐ODCu	n‐Propanol	17.9%	8.51 mA/cm^2^ &−0.94 V vs. RHE	150 h	[227]
	Cu_2_O/Ti_3_C_2_T_x_MXene	Propane	3.3%	− & −1.3 V vs. RHE	48 h	[228]
	Dendritic Cu‐Cu_2_O	Acetate	48%	4.6 mA/cm^2^ &−0.4 V vs. RHE	24	[204]
	Cu NCs	Propylene	14%	5.5 mA/cm^2^ &−0.6 V vs. RHE	16 h	[229]
High entropy alloy	CuMoRuFeW HEA	Ethylene	49.5%	250 mA/cm ^2^ & −1.1 V vs. RHE	14 h	[230]
	PdCuAuAgBiIn HEA Aerogels	Formic acid	98.1%	∼200 mA/cm ^2^ & −0.8 V vs. RHE	4 h	[209]
	AuAgPtPdCu HEA	Methane	49.4%	6.83 mA/cm^2^ at −0.7 V vs. Ag/AgCl	5 h	[208]
	Fe_0.1_Co_0.35_Ni0.35Cu_0.1_Mo_0.1_ HEA	Carbon monoxide	—	878 mA/cm^2^ at −1.5 V vs. RHE	110 h	[231]
	Cu‐rich CuBiInZnPd HEA	Formate	93.17%	150 mA/cm^2^ at −1.0 V vs. RHE	20 h	[232]
	BiSbInCdSn‐O_4_	Formate	98%	150 mA/cm^2^ at −0.3 V vs. RHE	100 h	[233]
	CuCoAlFeNi HEA	Ethanol	36.56%	102.4 mA/cm^2^ at −0.8 V vs. RHE	23 h	[207]
	AuAgCuPdPt HEA	Carbon monoxide	96.5%	194.2 mA/cm^2^ at −0.8 V vs. RHE	833 h	[234]
	perovskite La(FeCuMnMgTi)O_3_	C_2+_ products	92%	21.9 mA/cm^2^ at −0.70 V vs. SCE	5 h	[212]
Non—Cu system	ImF‐Mo3P	Propane	91%	395 mA/cm^2^ & −0.8 V vs. RHE	100 h	[215]
	SnS2/Sn1−O3G	Ethanol	82.5%	17.8 mA/cm^2^ & −0.9 V vs. RHE	100 h	[235]
	Ag‐G NCF	Ethanol	85.2%	0.31 mA/cm^2^ & −0.9 V vs. RHE	10 h	[236]
	NiP_2_	Methylglyoxal	84%	<0.5 mA/cm^2^ & −0.1 V vs. RHE	2.5 h	[237]
	SAM‐modified Au	Ethylene glycol	87%	∼−0.4 mA/cm^2^ & −0.58 V vs. RHE	8 h	[238]
	Nie‐(Cr_2_O_3_)_3_(Ga_2_O_3_)	1‐Butanol	42%	− & −1.48 V vs. Ag/AgCl	175 h	[216]
	Cr−Ga/GC	Oxalate	59%	8−10 mA/cm^2^ & −1.48 V vs. Ag/AgCl	240 h	[214]
	Fe_2_P_2_S_6_	Ethanol	23.1%	<0.5 mA/cm^2^ & −0.2 V vs. RHE	30 h	[239]
	Ru PC/NPC	Ethanol	27.5%	<1 mA/cm^2^ & −0.97 V vs. NHE	3 h	[240]
	NDD/Si RA	Acetate	91.8%	− & −1.0 V vs. RHE	3 h*10 batch	[241]
SAC	NiP−N_4_−C	Carbon monoxide	≥99%	200 mA/cm^2^ & −0.7 V vs. RHE	10 h	[162]
	MoSA−SeSA	Carbon monoxide	98.3%	24.5 mA/cm^2^ at −0.6 V vs. RHE	23 h	[242]
	Cu SAs/GDY	Methane	66%	24 mA/cm^2^ &−1.3 V vs. RHE	10 h	[243]
	Cu_99_In_1_	Carbon monoxide	91%	100 mA/cm^2^ & −0.7 V vs. RHE	12 h	[176]
	Cu_1_In_99_	Formic acid	91%	− & −1.2 V vs. RHE	—	[176]
	Cu/ceria‐H_2_	Methane	70.03%	− & 1.49 V vs. RHE	7.5 h	[244]
	Ni‐Fe DAC	Carbon monoxide	100%	608.2 mA/cm^2^ & −1.6 V vs. RHE	100 h	[245]
	rGO@CuN (H_x_) C	Methane	77.1%	163 mA/cm^2^ & −1.4 V vs. RHE	—	[246]
	De‐Au_1_Cu SAA	Ethylene	52%	252 mA/cm^2^ & −1.1 V vs. RHE	54 h	[247]
	Cu/C‐0.4	Ethanol	>90%	1.2 mA/cm^2^ & −0.7 V vs. RHE	16 h	[248]
Molecular catalyst	Fe‐TPP/Ni	Ethanol	58.2%	0.37 mA/cm^2^ & −0.3 V vs. RHE	62 h	[249]
	CuPc	C_2+_	70%	800 mA/cm^2^ & −0.73 V vs. RHE	10 h	[250]
	CoPc	Carbon monoxide	>90%	650 mA/cm^2^ & –	42 h	[251]
	Mn‐corrole	Acetic acid	63%	0.8 mA/cm^2^ & −0.674 V vs. RHE	5 h	[252]
	SnPc‐8F@CNTs	Formic acid	91.7%	440.6 mA/cm^2^ & −1.2 V vs. RHE	200 h	[253]
	Ni‐2D‐O‐SA‐ CNT	Methanol	27%	3.5 mA/cm^2^ & −0.9 vs. RHE	5.5 h	[254]
	CoPc/SWCNT	Methanol	53.4%	8.8 mA/cm^2^ & −0.93 V vs. RHE	10 h	[255]
	Hex‐2Cu‐O	n‐Propanol	16.7%	∼9.4 mA/cm^2^ & −1.3 V vs. RHE	25 h	[256]
	CuBr‐4PP	n‐Propanol	∼10% to 12%	—& −2.2 V vs. Ag/AgCl	6 h	[189]
	CuPc‐DFP‐4OH‐ Cu	Ethylene	56.86%	108.7 mA/cm^2^ & −0.7 V vs. RHE	60 h	[257]

## 5 Mapping the Reaction Pathways for Selectivity

Developing catalysts capable of driving the multi‐proton, multi‐electron transformation of CO_2_ is essential for converting it into value‐added products such as higher hydrocarbons and oxygenates. Achieving this level of chemical complexity, however, requires more than high activity; it demands precise control over the reaction pathway [261]. The catalyst must stabilize key intermediates, manage proton‐electron transfer sequences, and suppress competing paths to steer the reaction toward desired products [262]. Therefore, effective CO2RR catalyst design hinges not only on enabling demanding redox chemistry but also on strategically directing the mechanistic pathway to maximize selectivity and product value.

**FIGURE 11:**
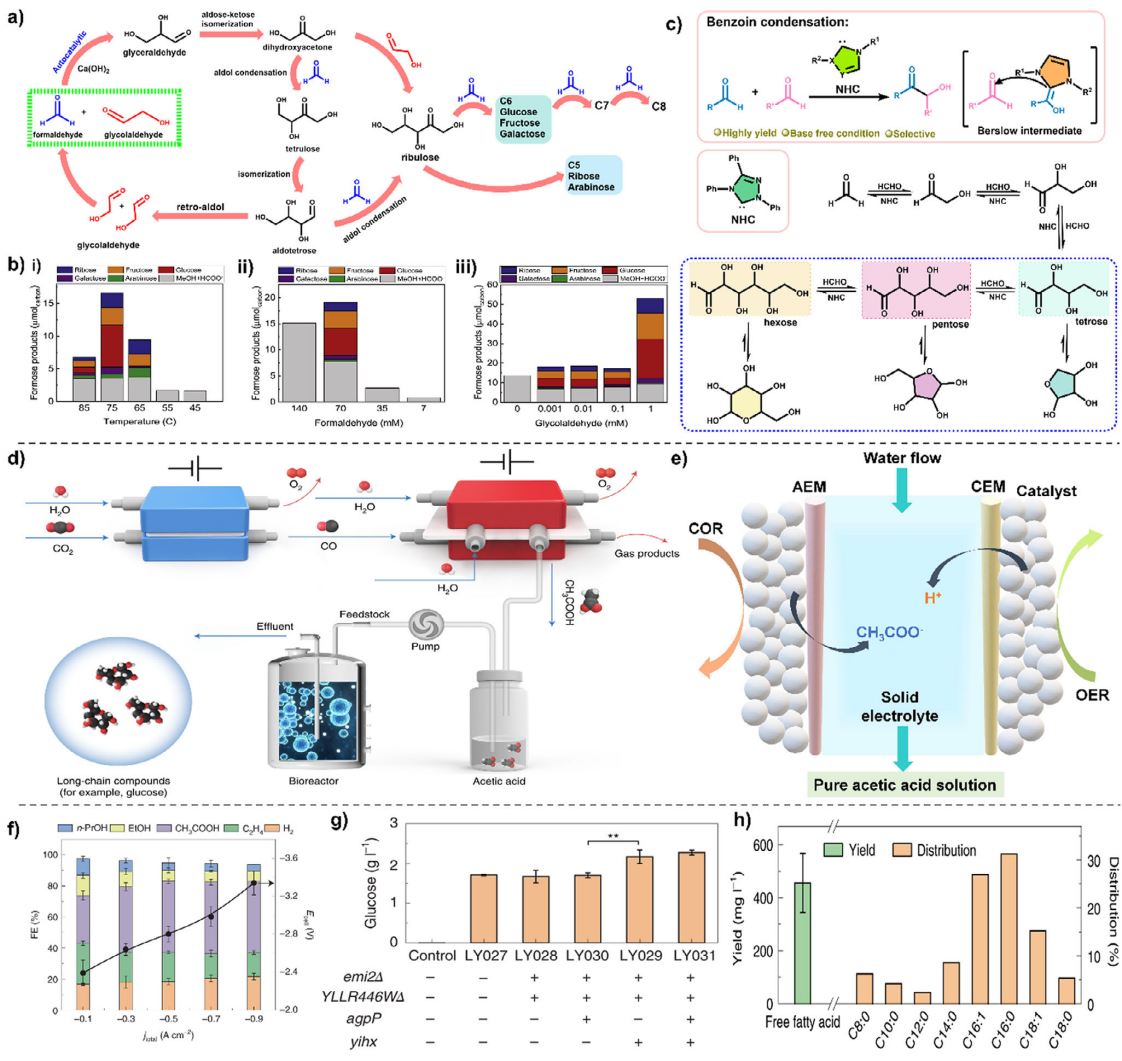
(a) Scheme illustrating the glycolaldehyde‐driven formose reaction, where formaldehyde is converted into C_3_‐C_6+_ carbohydrates. (b) Formose reaction product selectivity was assessed versus (i) temperature, (ii) formaldehyde, and (iii) glycolaldehyde concentrations, where carbohydrates were quantified by HPAEC‐PAD and methanol/formate by 1H NMR. Reproduced with permission from [258] Copyright 2022, Elsevier Inc. (c) Schematic representation of the NHC‐mediated formoin condensation mechanism, whereby iterative formoin couplings produce stable C_4_, C_5_, and C_6_ carbohydrate species. Reproduced with permission [259]. Copyrights 2025, National Academy of Sciences. (d) Conceptual schematic of an in vitro system where CO_2_ is electrochemically converted to acetic acid via a two‐step process and directly fed into a bioreactor for microbial conversion into higher‐order carbohydrates. (e) Schematic representation of CO reduction to high‐purity acetic acid in the solid‐electrolyte reactor, where AEM denotes the anion exchange membrane, CEM the cation exchange membrane, and OER the oxygen evolution reaction. (f) High‐purity acetic acid production on a GB‐Cu catalyst in a solid‐electrolyte reactor, with CO reduction FE and iR‐corrected cell voltages plotted against total current density. (g) Targeted genetic engineering of yeast to express a specific glucose‐1‐phosphatase significantly enhances glucose biosynthesis. (h) The genetically engineered yeast strain LXJ015 utilised acetic acid to biosynthesise free fatty acids with chain lengths from C_8_ to C_20_. Reproduced with permission from [260]. Copyright 2022, Nature Publishing Group.

The conversion of CO_2_ into complex carbohydrates is a flagship route for sustainable utilization of waste carbon. Although biological photosynthesis serves as the natural benchmark, its overall solar‐to‐chemical energy conversion efficiency is typically constrained to 0.1%–1% [263, 264]. Current research on abiotic CO_2_ reduction focuses on high‐rate inorganic catalysis to overcome the resource limits of conventional methods [265, 266, 267, 268]. Yang and coworkers proposed a foundational roadmap for abiotic CO_2_‐to‐sugar conversion, outlining formose‐ and formoin‐based strategies that differ in their molecular building blocks for producing life‐sustaining sugars [258, 259]. Given the advantages of electrochemistry over thermochemistry, such as milder reaction conditions and lower capital costs, in 2022, they pursued an electrocatalytic route to produce formaldehyde. They identified the formose reaction as a promising pathway for generating sugars from simple molecules. In the formose pathway, formaldehyde and glycolaldehyde serve as the primary substrates, undergoing sugar formation catalyzed by divalent Ca^2+^ ions at mildly elevated temperatures and alkaline conditions [269, 270]. Glycolaldehyde acts as an autocatalytic initiator (Figure 11a), suppressing the competing Cannizzaro disproportionation of formaldehyde and promoting aldol condensation, thereby enabling selective sugar production [271, 272, 273]. Figure 11b‐i shows that formose reaction optimization is governed by temperature, with activation above 55°C, a temperature‐driven shift from C_5_ sugars at 65°C to predominantly C_6_ sugars (∼78%) at 75°C, and thermal degradation at 85°C. Figure 11b‐ii and b(iii) indicate that efficient formose chemistry depends on an optimal formaldehyde concentration (∼70 mM) to favor C─C coupling over Cannizzaro disproportionation, [274] together with a minimum glycolaldehyde level (∼1 µM) required to initiate the autocatalytic initiator for synthesizing bio‐sustained sugars. Following established literature, they first utilized direct electrochemical CO_2_ reduction to generate glycolaldehyde on copper nanoparticle catalysts and formaldehyde on boron‐doped diamond electrodes (BDD) [275, 276]. Although the production of formaldehyde via BDD electrodes was identified as a major bottleneck in this work, owing to its poor stability in aqueous media and insufficient production rates to sustain the formose reaction, the formose pathway was nevertheless verified to proceed when trace amounts of glycolaldehyde were present [258]. A major drawback arises from the autocatalytic behavior of the system, which induces kinetic instability, hinders precise control over product selectivity, and consequently lowers carbohydrate carbon yields in the formose pathway. In this context, building blocks serve as the primary descriptors for sugar synthesis and are interconvertible through various abiotic processes [277, 278] More recently, they established a three‐step modular pathway to bypass the bottleneck associated with direct reduction. In this modular pathway, CO_2_ is first electrochemically hydrogenated to methanol over a CoPc/CNT catalyst, achieving a methanol partial current density exceeding 10 mA cm^−2^. Methanol is then selectively photocatalytically oxidized to formaldehyde using a zinc indium sulfide nanocrystal catalyst with photo‐deposited Ni, enabling concurrent hydrogen evolution. Finally, formaldehyde undergoes an Enders carbene‐mediated formoin reaction to enable abiotic sugar synthesis [259]. Formaldehyde serves as the primary substrate in benzoin‐type condensations, commonly referred to as the formoin reaction, which are catalyzed by N‐heterocyclic carbenes (NHCs) to generate C_2_‐C_4_ carbohydrates [279, 280, 281] 1,3,4‐triphenyl‐4,5‐dihydro‐ l,2,4‐triazol‐5‐ylide (NHC‐1) was employed under base‐free, non‐aqueous conditions using an alcohol‐trapped precursor that generates the active carbene in situ upon heating. This approach eliminates the need for external bases, which commonly induce aldose‐ketose isomerization and hinder the growth of linear multicarbon chains beyond C3 hydrocarbons [282]. Moreover, sugar selectivity is enhanced by the steric bias of the NHC‐1 catalyst, which preferentially engages formaldehyde to form the nucleophilic Breslow intermediate while suppressing competing C_2_‐C_2+_ coupling routes (Figure 11c). In contrast to conventional alkaline formose conditions, which produce broad product distributions, the NHC‐mediated system stabilizes ketoses, such as dihydroxyacetone, as terminal, unreactive species, thereby preventing the formation of toxic branched sugars [283]. The formoin strategy offers superior scalability by coupling high‐rate electrochemical CO_2_‐to‐methanol conversion with subsequent selective photocatalytic oxidation to formaldehyde, thereby circumventing the low throughput and separation challenges inherent to direct electrochemical formaldehyde production. Sugars generated via the formoin pathway exhibited enhanced biocompatibility, enabling E. coli cultures to reach substantially higher stationary‐phase optical densities (∼0.6 OD) compared to those supported by formose‐derived substrates (∼0.22 OD). This improvement stems from the selective formation of metabolically accessible, energy‐rich monosaccharides, free of the inhibitory branched isomers typically produced in conventional formose chemistry.

Furthermore, Zheng et al. introduced a hybrid electro‐biosystem by spatially integrating decoupled CO_2_ electroreduction with microbial fermentation to selectively produce long‐chain hydrocarbons. A schematic depiction of the in vitro artificial sugar synthesis system is shown in Figure 11d, wherein electrochemically synthesized high‐purity acetic acid serves as the carbon feedstock for yeast fermentation. Decoupled electrolysis keeps bio‐integrated microbial systems working steadily even at high current densities. It also shields microbes from the harsh, toxic conditions in traditional electrolyzers, where product streams mix with concentrated electrolyte salts that quickly kill the microbes through apoptosis [284]. They chose a two‐step CO_2_ electroreduction pathway over direct CO_2_‐to‐acetate conversion to replace the expensive Monsanto process for making acetic acid [285] due to the latter's poor selectivity and productivity [240, 286, 287] Here Ni─N─C single‐atom catalyst produces CO nearly 100% efficiently (FE_CO_ ∼100%, j_CO_ −154 mA cm^−2^), [288] overcoming low CO* coverage that limits C─C coupling [289] on Cu for CH_3_COOH production. Then the grain‐boundary‐rich Cu (GB‐Cu) beats planar or annealed GB‐Cu by lowering the energy barrier for CO* + H* → HCO*, enabling favorable C‐C coupling to CHCO* [290, 291] Cu (100)/ (111) facets favor CHCO* → CHCHO* over CH_2_CO*, yielding selective acetic acid over C_2+_ alcohols in a solid electrolyte system [44]. Finally, a solid‐electrolyte reactor produces bio‐sustained pure CH_3_COOH feedstock by electrically driving CH_3_COO^−^ from the cathode and H^+^ from the anode across ion‐exchange membranes into a central porous layer (Figure 11e). This captures the purification mechanism in one sentence, emphasising clean acetate protonation for microbial use.

Acetic acid purity exceeds 90 wt.% through concentration‐gradient transport and electroosmotic drag of alcohol by‐products into the porous solid‐electrolyte layer (Figure 11e). A thick anion‐exchange membrane blocks alcohol crossover, enabling continuous ∼97 wt.% production of ultrapure acetic acid (Figure 11f) for 140 h at −250 mA cm^−2^. Yeast (*S. cerevisiae*) converts electrochemically produced acetic acid from CO_2_ into fermentable sugars in an integrated bio‐system. Acetic acid replaces ethanol as the substrate, since both funnel into acetyl‐CoA in yeast metabolism. This highlights the metabolic pivot for sustainable sugar production from CO_2_‐derived feedstocks [292, 293]. Acetyl‐CoA from CO_2_‐derived acetic acid feeds the glyoxylate cycle, yielding oxaloacetate for gluconeogenesis into glucose phosphates (G6P, G1P) and glucose. Yeast naturally glycolyzes glucose via hexokinase phosphorylation of glucose to G6P, so deleting glk1Δ, hxk1Δ, and hxk2Δ genes creates a “glucose leaky” strain that produces but does not consume its own sugars. Native yeast phosphatases enable glucose secretion, but Figure 11g shows targeted boosts work best: deleting extra hexokinases or expressing AgpP alone has little effect, while *E. coli* G1P‐specific phosphatase YihX (in LY029/LY031 strains) sharply increases glucose output. This clarifies the engineering tweaks to improve sugar yields from CO2 feedstocks without overcomplicating the roles of phosphatases. Further, the process demonstrates that strain LY031 efficiently converts acetic acid directly into glucose (∼1.81 g L^−1^), avoiding salt‐induced cytotoxicity common in conventional flow‐cell systems, while Figure 11h highlights platform versatility by enabling strain LXJ015 to produce ∼500 mg L^−1^ of C_8_‐C_20_ saturated free fatty acids from the same acetate feedstock [260].

## 6 Summary and Outlook

**SCHEME 1:**
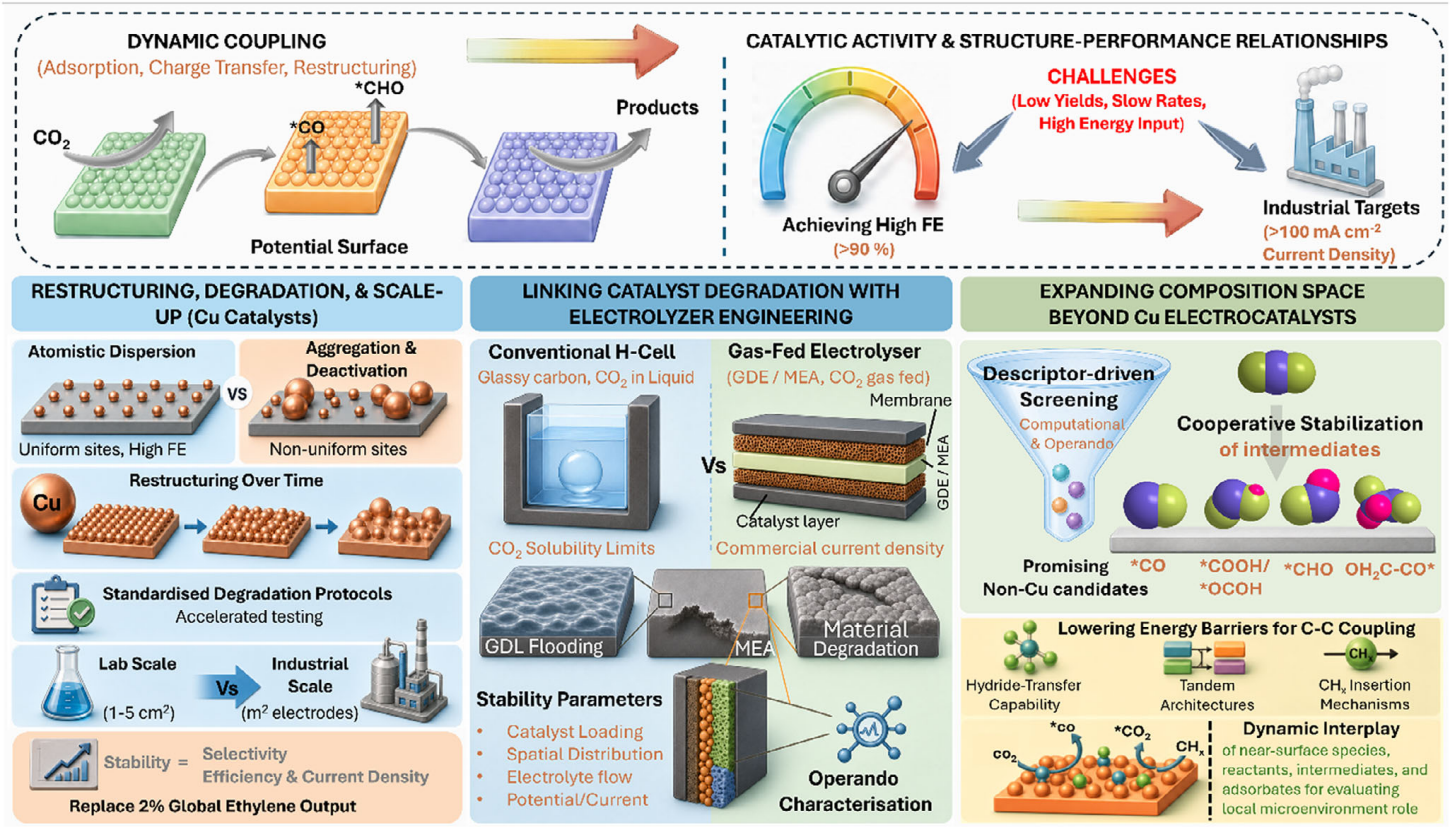
Overview of heterogeneous electrocatalysis for selective CO_2_RR and the way forward.

The functionality of heterogeneous catalysis is rooted in the dynamic coupling of adsorption, charge transfer, and catalyst restructuring at solid‐gas and solid‐liquid interfaces. Hence, elucidating catalytic activity and establishing clear structure‐performance relationships are central to mechanism‐guided catalyst design, enabling the rational and efficient creation of advanced catalytic materials (Scheme 1). In this review, we provide a foundational insight probing the potential of different electrocatalysts in selective CO_2_RR to yield an array of high‐value products. This unlocks unprecedented efficiency and versatility for sustainable carbon utilization. We realize that product selectivity is dictated by intermediate interactions, CO_2_ binding modes, applied potential, and adsorbate stability. Catalyst features, such as surface facets, morphology, surface reconstruction, and local microenvironment, further influence their thermodynamics and kinetics. So, a coordinated, interdisciplinary effort involving the application of advanced operando characterization and computational techniques is essential to investigate the CO_2_RR under actual operating conditions. Despite significant progress in product selectivity and efficiency of over 90%, CO_2_ electroreduction still faces significant challenges, including low yields, slow rates, and high energy demands. For instance, industrial‐scale CO_2_R requires electrolyzers capable of stable, efficient operation at current densities exceeding 100 mA cm^−2^, thereby impeding their commercialization. In conclusion, we emphasize several key issues that should be prioritized in future research.

### 6.1 Restructuring, Degradation and Scale‐Up Challenges of Copper Catalysts

Stability in CO_2_RR relies on maintaining atomically dispersed catalysts, as these configurations eliminate the possibility of aggregation, which often leads to deactivation. This same principle also governs selectivity: when every active site is identical and isolated, the system enforces a uniform reaction pathway, favoring the formation of a single product with remarkably high F.E. Based on the literature, we conclude that all Cu‐based catalysts undergo restructuring, albeit on different timescales [275, 294]. The next step is to establish standardized degradation protocols for benchmarking catalyst stability. Conducting long‐term tests under realistic operating conditions is often impractical in academic settings, making accelerated testing essential. These tests should be designed around the key driving forces behind catalyst degradation and reconstruction. We argue that stability metrics should be given equal emphasis alongside efficiency, selectivity, and current density. Also, although morphological, electronic, and interfacial engineering of Cu catalysts has enabled C_2+_ products with FE above 90% at >1 A cm^−2^, nearing economic viability, this has been achieved only at the laboratory scale (1–5 cm^2^) [43, 235, 295]. Replacing 2% of global ethylene output would demand ∼15 GW of capacity and >10 tonnes of catalyst annually. Addressing this demand requires catalysts that maintain laboratory‐scale activity and selectivity upon kilogram‐scale production and coating over square‐meter electrodes.

### 6.2 Linking Catalyst Degradation Mechanisms With Electrolyzer Engineering

The integration of carbon‐based gas diffusion layers (GDLs) and catalyst layers into membrane electrode assemblies (MEAs) has attracted significant attention as research increasingly shifts toward gas‐fed electrolyzer configurations [296, 297]. These systems overcome the solubility limitations of CO_2_ in aqueous electrolytes by directly feeding gaseous CO_2_ to the catalyst interface, thereby achieving commercially relevant current densities. Compared to conventional H‐cell setups, gas‐fed electrolyzers typically operate at lower overpotentials and achieve higher local pH conditions, facilitating more efficient CO_2_ electroreduction. Translating catalyst degradation mechanisms and mitigation strategies from the well‐controlled environment of an H‐cell to gas‐fed electrolyzers remains a major challenge. Beyond the intrinsic differences in operating conditions, practical engineering issues, such as GDL flooding and GDL material degradation, must also be addressed. Compared to the extensively studied H‐cell configuration, in which catalysts are typically supported on flat glassy carbon electrodes, the understanding of stability‐determining parameters in gas diffusion electrode (GDE) and MEA systems remains limited. Critical factors such as catalyst loading, spatial distribution within the GDL, electrolyte composition and flow rate, electrolyte phase (liquid or solid), and applied potential or current all demand systematic investigation. Moreover, advancing this field requires the development of tailored in situ and *operando* characterization techniques, as the catalytic interface in GDE‐ and MEA‐based setups is far less accessible to conventional surface analysis compared to planar electrodes.

### 6.3 Expanding the Compositional Space beyond Cu Electrocatalysts

High selectivity toward C_4+_ products at current densities exceeding 100 mA cm^−2^ depends on a coordinated catalyst and electrode design. Metals with strong hydrogen‐binding characteristics, such as Ni and Pd, promote the formation of surface CH_x_ intermediates and facilitate subsequent C‐C bond formation at low overpotentials. Anchoring these active sites onto durable carbon supports helps maintain catalytic stability while suppressing undesired reaction pathways. In parallel, electrode architectures incorporating tailored porosity and ionomer‐integrated or gas‐diffusion configurations improve mass transport and sustain high reaction rates. Together, these synergistic strategies establish a practical framework for directing CO_2_ conversion toward deeply reduced, multicarbon products. Systematic screening of non‐Cu catalyst combinations should be guided by mechanistic descriptors rather than by empirical trial‐and‐error. Promising candidates are those that can cooperatively stabilize key C_1_ intermediates (*CO, *CHO, *COOH, or *H_2_CO) while simultaneously lowering the energy barrier for sequential C‐C coupling and chain propagation, particularly through hydride‐assisted or formaldehyde‐mediated pathways [214, 298]. Emphasis should be placed on materials exhibiting hydride‐transfer capability and multicenter cooperativity, as demonstrated in Ni_2_P systems enabling C_4_ product formation [214], as well as tandem architectures that separate intermediate generation and downstream coupling steps [216, 299]. Bimetallic or hybrid systems should be evaluated for complementary adsorption strengths to maintain optimal intermediate surface coverage without overbinding, consistent with Fischer‐Tropsch‐like CHx insertion mechanisms observed on polarized Ni systems [220]. In addition, ion‐doping or electronic modulation strategies that stabilize *CO/*CHO intermediates can further enhance C‐C coupling selectivity [300], Computational screening of intermediate binding energies and coupling energetics, combined with operando validation under high current densities, can efficiently narrow the compositional space. Such a descriptor‐driven strategy enables rational identification of robust non‐Cu catalyst assemblies capable of sustaining selective C_4+_ production at practical reaction rates. The role of the microenvironment in governing CO_2_R should be evaluated alongside the catalyst's evolving state. A comprehensive understanding requires accounting for the dynamic interplay among near‐surface species, reactants, intermediates, and adsorbates to effectively identify the key factors that steer the reaction toward desired pathways to produce transportation fuels and essential commodity chemicals.

## Author Contributions

**Julia Wiktor**: supervision, software, visualization, investigation, writing – original draft. **Bapan Biswas**: conceptualization, methodology, validation, investigation, writing – original draft, writing – review and editing, visualization. **Ujjwal Pal**: supervision, conceptualization, investigation, writing – original draft, writing – review and editing, validation, project administration. **Suvodeep Sen**: conceptualization, investigation, writing – original draft, methodology, validation, visualization, writing – review and editing. **Apinya Ngoipala**: conceptualization, writing – original draft, validation, investigation, software, visualization. **Shalini Singh**: conceptualization, investigation, writing – original draft, writing – review and editing, validation, methodology, supervision, funding acquisition, project administration.

## Conflicts of Interest

The authors declare no conflict of interest.

## Data Availability

The data that support the findings of this study are available from the corresponding author upon reasonable request
